# Interfacial Water Configurations: Evolution and Implications for Electrocatalysis

**DOI:** 10.1002/advs.76907

**Published:** 2026-07-29

**Authors:** Aoqi Wang, Yang Song, Lin Guo, Chenchen Weng, Yanxia Yuan, Chaoyu Li, Xue Yang, Wei Lin

**Affiliations:** ^1^ SINOPEC Research Institute of Petroleum Processing Beijing China; ^2^ State Key Laboratory of Petroleum Molecular and Process Engineering Beijing China

**Keywords:** catalysis, configurations, electrochemistry, interfacial water

## Abstract

Water plays a pivotal role in electrocatalysis, extending far beyond its function as a solvent to govern proton‐coupled electron transfer and interfacial mass transport. The high O─H bond energy, however, renders water dissociation a major kinetic bottleneck in many electrocatalytic reactions. The configurational details of the interfacial water layer on catalyst surfaces, particularly molecular orientation and hydrogen‐bonding networks within the electrical double layer under applied bias, govern the electrochemical performance of electrode materials. In catalytic processes, they facilitate the transport of protons, electrons, and other reactive species among diverse active sites and ultimately enhance reaction kinetics. Despite the significant impact of interfacial water, deciphering its configurations and translating this understanding into rational control over catalytic processes remains elusive. This review addresses this critical challenge by providing a comprehensive and mechanistic understanding of how interfacial water structures govern reaction pathways. We aim to bridge the gap between unraveling the intricate role of water structures and establishing robust design principles for advanced catalysts. Thus, this work provides a transformative foundation for developing more efficient and selective electrocatalysts, ultimately enabling enhanced control over catalytic outcomes.

## Graphical abstract

A comprehensive and mechanistic understanding of how interfacial water structures govern reaction pathways. This review aims to bridge the gap between unraveling the intricate role of water structures and establishing robust design principles for advanced catalysts. As a result, it provides a transformative foundation for developing more efficient and selective electrocatalysts, ultimately enabling enhanced control over catalytic outcomes.

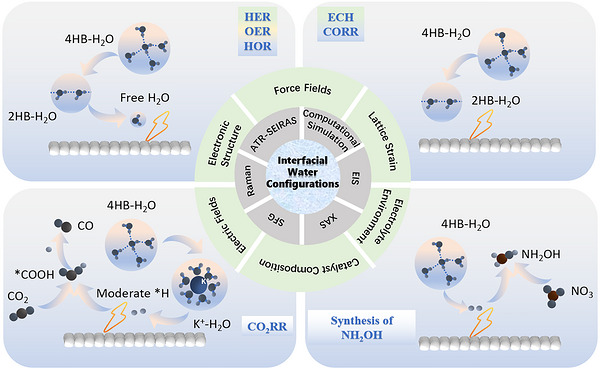

## 1 Introduction

Among Earth's natural cycles, four elements‐carbon, hydrogen, oxygen, and nitrogen‐constitute the fundamental building blocks of energy storage and conversion systems [1]. Circulating continuously through the atmosphere, hydrosphere, and geosphere, these elements drive the planet's energy balance and sustain the technological applications that underpin modern society. From an energy perspective, the chemical bonds formed between them, particularly N─H, H─O, and C─H bonds, serve as critical energy carriers [2, 3]. The ability to efficiently break and reform bonds such as H─O in water splitting and N─H in ammonia synthesis or decomposition underpins a diverse array of key electrochemical processes. This ability directly determines the performance of electrocatalytic devices, including water electrolyzers, fuel cells, CO_2_ reduction systems, electrochemical hydrogenation (ECH), and ammonia‐to‐hydrogen converters [4, 5, 6, 7, 8]. Catalytic technology is therefore central to energy conversion, green chemical engineering, and environmental remediation. Despite longstanding efforts to understand the electronic and geometric structures of solid catalysts, most industrially relevant catalytic processes take place at heterogeneous interfaces, particularly in liquid‐phase systems [9, 10, 11]. Consequently, the efficacy and stability of electrocatalytic systems are governed not only by the intrinsic properties of the catalyst but also by molecular‐scale dynamics at the gas‐liquid‐solid interface [12].

**FIGURE 1:**
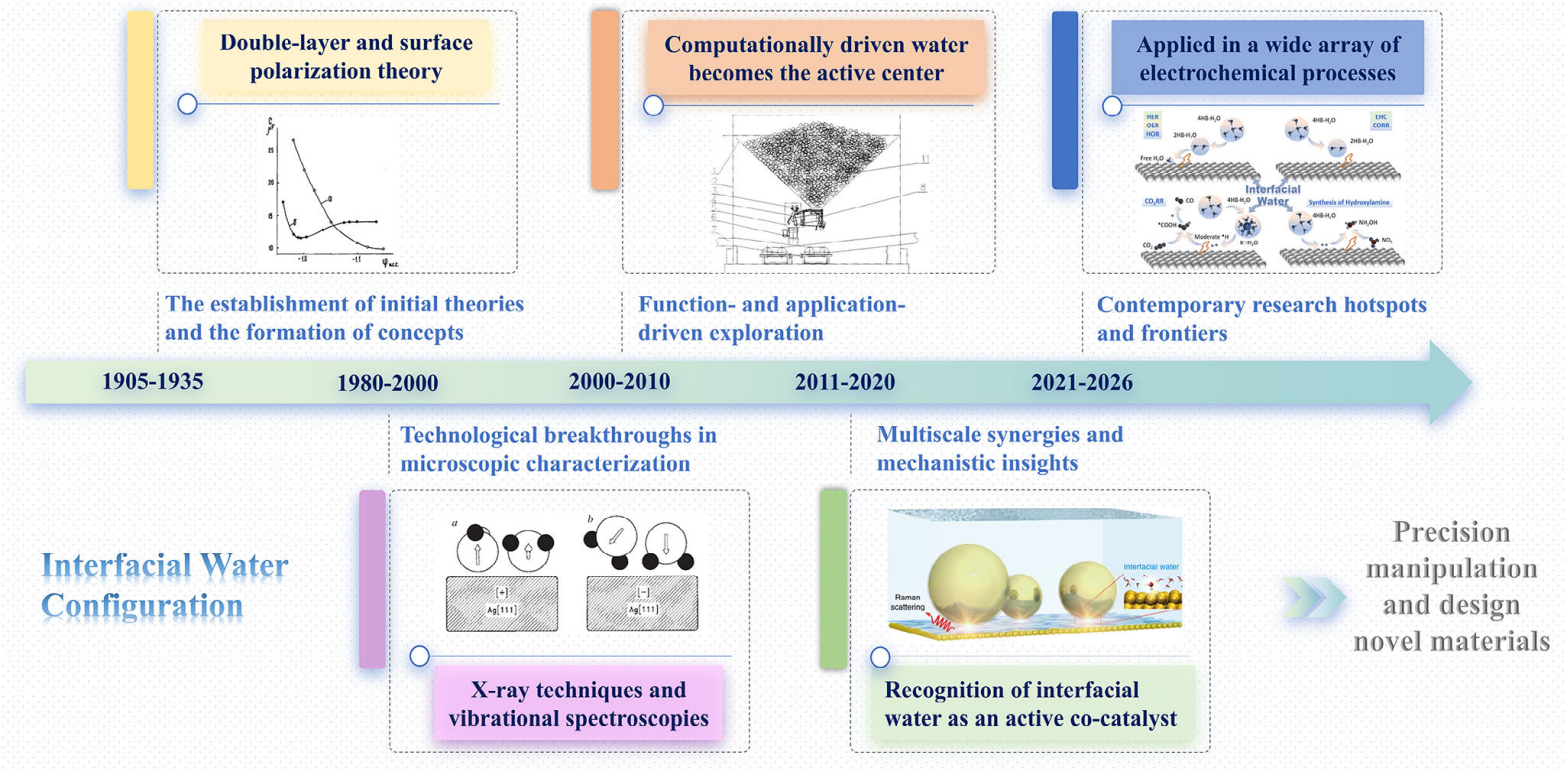
Timeline of key milestones in the development of interfacial water research in electrocatalysis.

Within this context, water, as the natural carrier of hydrogen and oxygen, occupies a central position [13, 14]. It functions as both reactant and medium in nearly all electrochemical energy conversion technologies [6, 15, 16]. At the three‐phase boundary, water forms a non‐uniform interfacial region that differs markedly from the homogeneous bulk phase [17], where molecular‐scale dynamics, along with the inherent design of the catalyst ultimately dictate the efficacy and stability of electrocatalytic systems [18, 19]. To gain insight into these molecular‐scale dynamics, an in‐depth study of interfacial water is required. Figure 1 presents a concise timeline of key milestones in the development of interfacial water research in electrocatalysis, along with representative images from pivotal studies. The concept of water orientation at the electrode interface originated from early electrical double‐layer theories and surface polarization models proposed between 1905 and 1935 [20]. Pioneers such as Frumkin established quantitative relationships between reaction rates and interfacial structure, laying the groundwork for later studies of water's structural impact [21]. With the application of radiation source‐based X‐ray techniques, infrared absorption spectroscopy (IRAS), Raman spectroscopy, and sum frequency generation (SFG), a preliminary model of the interfacial water structure was established [22, 23, 24, 25]. Between 2000 and 2010, the integration of density functional theory (DFT) and ab initio molecular dynamics (AIMD) enabled atomic‐scale simulation of water behavior, deepening the understanding of water configurations [26]. In the following decade, researchers began to view interfacial water not merely as a solvent but as an active co‐catalyst. Interfacial water was reported to facilitate the transport of protons, electrons, and other reactive species among diverse active sites and thereby enhance reaction kinetics [27]. The pivotal role of interfacial water is perhaps most evident in water electrolysis, where its structural dynamics and hydrogen‐bonding networks directly dictate reaction kinetics and overall system performance [28]. For example, Li et al. demonstrated that cation‐regulated water structures significantly influence the kinetics of the hydrogen evolution reaction (HER) using in situ shell‐isolated nanoparticle‐enhanced Raman spectroscopy (SHINERS) [29]. This period marked a shift from studying static water structures toward understanding their dynamic, functional roles in water splitting. The configurations of interfacial water, which govern the dynamic behavior, were characterized by ordered orientation and hydrogen‐bond networks in this period [29]. Since 2021, water configurations influence extends far beyond, profoundly shaping the competitive landscape in a wide array of electrochemical processes. They have been shown to impact a wide range of reactions and have emerged as a research hotspot and frontier [6, 12, 18, 30, 31]. In the HER, water molecules are reduced to generate molecular hydrogen. In the oxygen evolution reaction (OER), water is oxidized to produce oxygen. In the oxygen reduction reaction (ORR), water is the final product. Even in the CO_2_ reduction reaction (CO_2_RR), nitrogen reduction reactions, ECH, and hydroxylamine (NH_2_OH) synthesis, water serves as the proton source, directly influencing reaction pathways and product selectivity. During these reactions, the configurations of water are highly sensitive to factors such as electrode potential, pH, and surface chemistry, undergoing transient or steady‐state changes [32]. These changes allow water to directly participate in key steps, including proton‐coupled electron transfer, intermediate stabilization, and mass transport, thereby governing catalytic rate and pathway selectivity [12].

As the role of interfacial water has come to light, it has become evident that modulating the interfacial electric fields [15], force fields [33], and electrolyte environment [12]—factors that dictate water configuration—can yield catalytic processes with enhanced activity and selectivity. Interfacial water is therefore now widely recognized as a key factor governing reaction performance. Here, steering the interfacial water configuration is not merely beneficial but critical for tipping the balance between desired and parasitic pathways, thereby governing product selectivity and process efficiency [34]. This raises a fundamental question: Can we extend our inquiry beyond observation into the realms of active control and manipulation of these phenomena? Addressing this requires deciphering the governing principles of water dynamics and establishing robust structure‐activity relationships. Therefore, a thorough review about the advanced progress in interfacial water is of important significance, which sheds light on the relationship between property, structure, dynamic behavior of interfacial water, and reaction activity. This represents the frontier, an essential step toward transitioning from empirical catalyst design to atomic‐level optimization, ultimately breaking through the current efficiency limits that constrain the field.

This review systematically summarizes recent key advances in the understanding of water configurations within catalyst systems. A fundamental understanding of interfacial water is briefly introduced. State‐of‐the‐art characterization techniques are highlighted, including in situ vibrational spectroscopy, X‐ray‐based methods, and surface‐enhanced spectroscopy, as well as theoretical approaches such as molecular dynamics (MD) simulations and first‐principles calculations. Moving beyond a mere catalog of observations, we critically analyze how dynamically evolving water architectures govern reaction pathways in practical catalytic applications. By identifying universal patterns in interfacial water reorganization and deciphering their specific mechanistic roles across a spectrum of key reactions, this work aims to establish a foundational framework. Ultimately, it provides a transformative perspective: We focus on the rational engineering of the interfacial aqueous microenvironment, treating it as an integral element rather than a secondary concern, to achieve unprecedented catalytic performance. Our goal is to drive a paradigm shift in which the regulation of water is regarded as equally essential as the design of the electrocatalyst itself.

## 2 Understanding of Interfacial Water

### 2.1 Configurations of Interfacial Water

**FIGURE 2:**
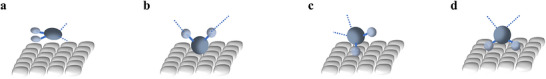
Schematic diagram of the interface water in different configurations. Interfacial water at surfaces that are (a) uncharged, (b) positively charged, (c) negatively charged, and (d) more negatively charged.

Hydrogen‐bonding interactions among water molecules endow them with a distinctive dynamic network architecture [35]. In disparate surface and interfacial environments, such interactions are markedly modulated by surface physicochemical properties, thereby inducing water molecules to adopt specific spatial configurations and organizational patterns. Based on variations in interfacial charge characteristics, interfacial water configurations can be broadly categorized into four archetypal scenarios, as depicted in Figure 2. On surfaces with negligible electrostatic heterogeneity (Figure 2a), the preferred adsorption geometry of water is typically characterized by a parallel alignment of its molecular plane relative to the surface, leading to the lowest energy state. For positively charged surfaces (Figure 2b), the oxygen atoms of water molecules, driven by their local electronegativity, orient toward the surface, yielding an ordered arrangement with oxygen termini pointing to the interface. In contrast, under the influence of a negatively charged surface (Figure 2c), water dipoles orient their positive ends (hydrogen atoms) toward the interface, thereby minimizing the electrostatic energy via the charge‐dipole interactions. As the surface negative charge density increases (Figure 2d), water molecules adopt a configuration where both O─H bonds point toward the surface, resulting in a strongly polarized interfacial water network. This phenomenon highlights the potent regulatory effect of interfacial electric fields on molecular orientation. Serving as both an intuitive illustration of charge‐driven water alignment and a fundamental basis for deeper inquiry, such configurational variations are essential for understanding the structure, dynamics, and functional behavior of interfacial water in physical, chemical, and biological systems.

### 2.2 Origins of Different Interfacial Water Configurations

**FIGURE 3:**
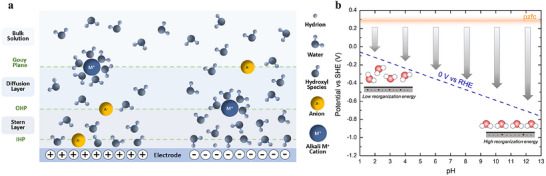
(a) The overall model of the EDL composed of solvent molecules, counterions, and specifically adsorbed co‐ions. (b) Pourbaix diagram showing the pH dependence of the hydrogen equilibrium potential (0 V vs. RHE), the effective PZC of Pt (111), and the water orientation and reorganization energy at the electrode/electrolyte interface. References with permission from Ref. [44], Copyright 2020, American Chemical Society.

Understanding the origin of distinct water configurations requires a thorough knowledge of the electric double layer (EDL) and its capacitance behavior [36, 37]. Upon contact between an electrode and a solution, an EDL forms at the interface between the two phases. Inner‐sphere reactions occurring on the electrode surface involve direct bonding of reactants to surface sites. The interconversion between electrical and chemical energy not only activates the electrode surface but also drives electron‐transfer processes [38]. As such, the molecular‐scale local environment of the electrode surface directly determines the reactive cross‐section of the reaction [39]. As illustrated in Figure 3a, the electric double‐layer comprises a Stern compact layer and an outer diffuse layer [40]. The Stern compact layer consists of the inner Helmholtz plane (IHP), which serves as the region of specific adsorption species, and the outer Helmholtz plane (OHP), which is formed by hydrated ions. The distribution of surface charge on the electrode induces a reorganization of charged particles at the interface, creating an EDL that differs substantially from that of the bulk electrolyte. A pronounced potential gradient exists within this interfacial region, with electric field strengths reaching approximately 10^8^ V m^−1^ [39]. Qualitatively, such an intense electric field can induce solvent ordering, align dipolar species, and promote ion accumulation at the interface, thereby modifying the free‐energy landscape of electrocatalytic reactions [41]. In the hydrogen adsorption process, H^+^ in acidic electrolytes and OH^−^ in alkaline electrolytes must transport across the EDL. The rate of charge transfer is critically dependent on the facilitative role of interfacial water molecules, which in turn is governed by the extent of structural reorganization at the interface [28].

However, the current understanding of the molecular structure of the EDL in certain reaction systems (e.g., platinum surfaces) remains inadequate, partly due to the existing uncertainty regarding their Potential of Zero Charge (PZC). The PZC of a metal refers to the electrode potential at which the metal surface carries no net charge when the metal is in contact with an electrolyte [42]. Variations in PZC are tightly correlated with alterations in the microenvironment of the EDL, which in turn directly modulate the dissociation of reactants, adsorption/desorption of intermediate species, and mass transport processes, thereby limiting the efficiency of charge transfer. Thus, for water‐involved electrochemical reactions, an in‐depth elucidation of the EDL structure on electrode surfaces provides a pivotal entry point for unraveling the kinetics of water dissociation. Studies demonstrate that at the PZC potential, water molecules adopt a planar parallel configuration on the surface (Figure 2a). At potentials above PZC, water molecules preferentially orient with their oxygen atoms pointing toward the surface (Figure 2b). When the potential is lowered below PZC, the orientation shifts so that the hydrogen atoms face downward (Figure 2c). With a further decrease in potential, a configuration in which both hydrogen atoms are directed toward the surface can also emerge (Figure 2d) [43]. Furthermore, the pH‐dependent HER or hydrogen oxidation reaction (HOR) activities are also related to PZC. Figure 3b shows that the PZC increases with pH, which leads to a higher negative charge density at the reactive surface, thus favoring an H‐down orientation [44].

Therefore, determining the PZC is essential for comprehending interfacial water configurations. Experimentally, the PZC can be identified through analysis of cyclic voltammetry (CV) curves. Theoretically, Gouy‐Chapman (GC) double‐layer theory predicts that the differential capacitance (Cd) reaches a minimum at the PZC, provided it falls within the interfacial potential window [42]. Notably, the laser‐induced temperature jump methods offer a quantitative route to PZC. However, their validity requires interfacial water reconfiguration to dominate the entropy response, a condition often violated in the presence of adsorbates. To overcome this limitation, Xu et al. developed an approach based on electric field‐induced second‐harmonic generation (SHG) [45]. Alternatively, other methodologies, such as CO displacement and persulfate reduction experiments, have been employed by researchers to determine PZC [42, 46, 47].

However, obtaining PZC only provides an inference of the evolution at the reaction interface from the perspective of the EDL theory, serving as a fundamental clue to changes in water configuration. As scientific research delves deeper into understanding this process, researchers are eager to “observe” the structural transformations of water at the molecular or even atomic scale. This has motivated the development of a suite of advanced characterization techniques. For instance, the qplus atomic force microscope enables the “visualization” of hydrated protons [48, 49], albeit under demanding conditions such as ultra‐high vacuum and low temperatures, which impose strict limitations on applicable substrates and surface states. To complement such approaches and probe water under operando conditions, vibrational spectroscopy is widely applied to investigate liquid‐phase water, as the O─H stretching frequency serves as a highly sensitive probe of intermolecular distance [50]. Furthermore, X‐ray‐based techniques (absorption, emission, and scattering) provide insights into the inner‐shell electrons of atoms [51, 52], while multiscale computational simulations and machine learning (ML) [53], offer robust support for elucidating the evolution of water configurations during catalytic processes.

## 3 Approaches to Investigating Water Molecular Configurations

### 3.1 Surface‐Enhanced Infrared Absorption Spectroscopy Combined With Attenuated‐Total‐Reflection Technique (ATR‐SEIRAS)

**FIGURE 4:**
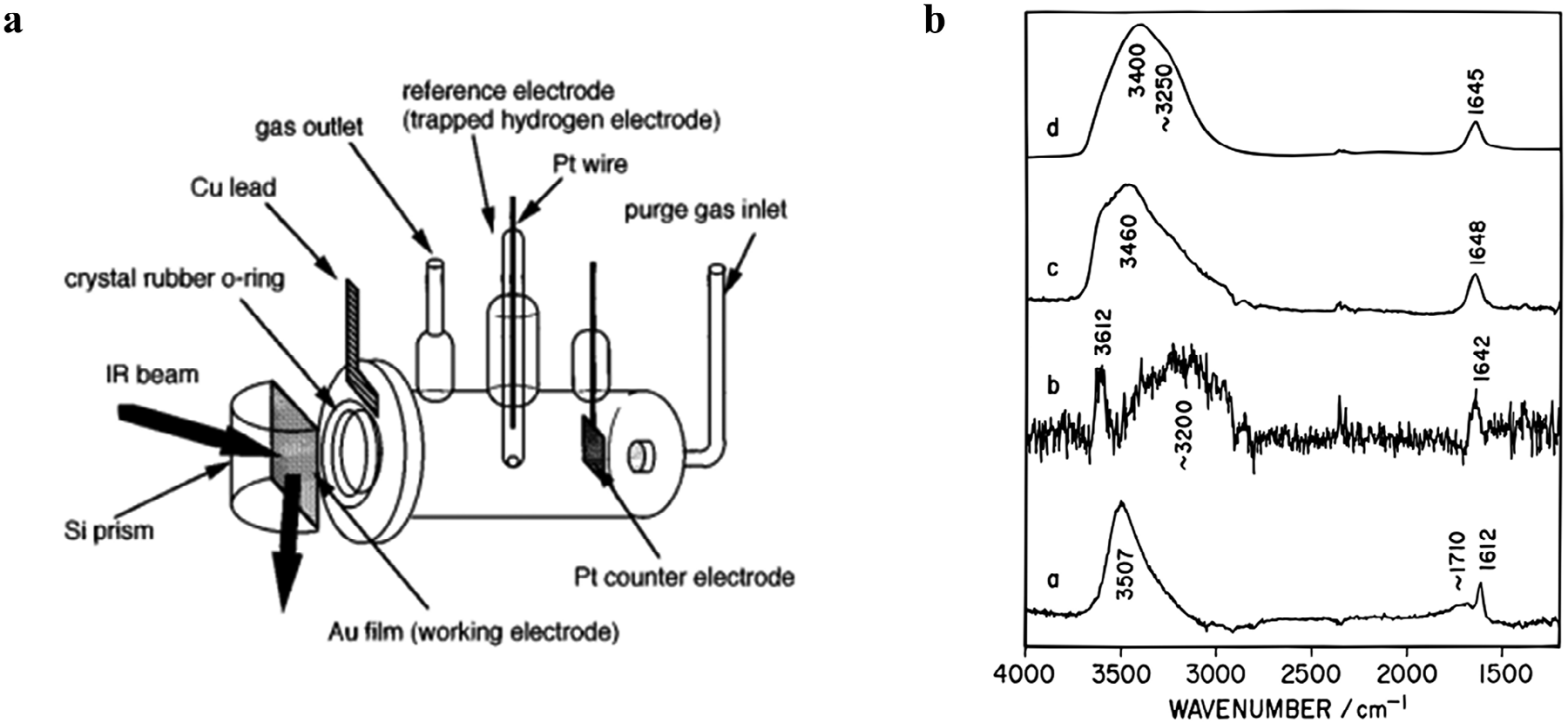
(a) Schematic diagram of the ATR‐SEIRAS cell. (b) Comparative ATR‐SEIRAS spectra of interfacial water on an Au (111) electrode in 0.5 M HClO_4_ at three different applied potentials: 0.12 V (curve a), 0.77 V (curve b), and 1.22 V (curve c), alongside the corresponding transmission spectrum of bulk water (curve d) for reference. Reproduced with permission from Ref. [23]. Copyright 1996, American Chemical Society.

ATR‐SEIRAS offers high‐sensitivity detection of vibrational signals from interfacial water molecules and is widely employed to study their potential‐dependent reconfigurations [23, 54]. The signal enhancement stems from the electric field generated by localized surface plasmon resonance in a thin gold film deposited on the electrode, which confines the probing depth to a few molecular layers near the surface. This configuration effectively suppresses interference from the bulk electrolyte while enabling rapid data acquisition with high signal‐to‐noise ratios. A schematic of the spectroscopic cell used in such measurements is presented in Figure 4a [23].

ATR‐SEIRAS characterization results demonstrated that at least three distinct configurations of water molecules existed at the interface, with their relative intensities varying in response to potential changes. A marked alteration in water configuration was observed when the potential deviated from the PZC. Specifically, at potentials below PZC, the O─H stretching vibration band (3000–3600 cm^−1^) exhibited a blueshift (Figure 4b, curve a) relative to bulk water (Figure 4b, curve d), while the H─O─H bending vibration band (1610–1650 cm^−1^) underwent a redshift compared with bulk water, accompanied by a narrowed full width at half maximum (FWHM). These spectral features indicated weakened hydrogen bonding among water molecules and a configurational transition of water from a hydrogen donor to a hydrogen acceptor. In contrast, at potentials above PZC, the O─H stretching vibration band broadened and shifted to lower frequencies (Figure 4b, curve c); the emergence of an “ice‐like” water structure with multiple hydrogen bonds implied a stronger hydrogen‐bonding interaction.

**FIGURE 5:**
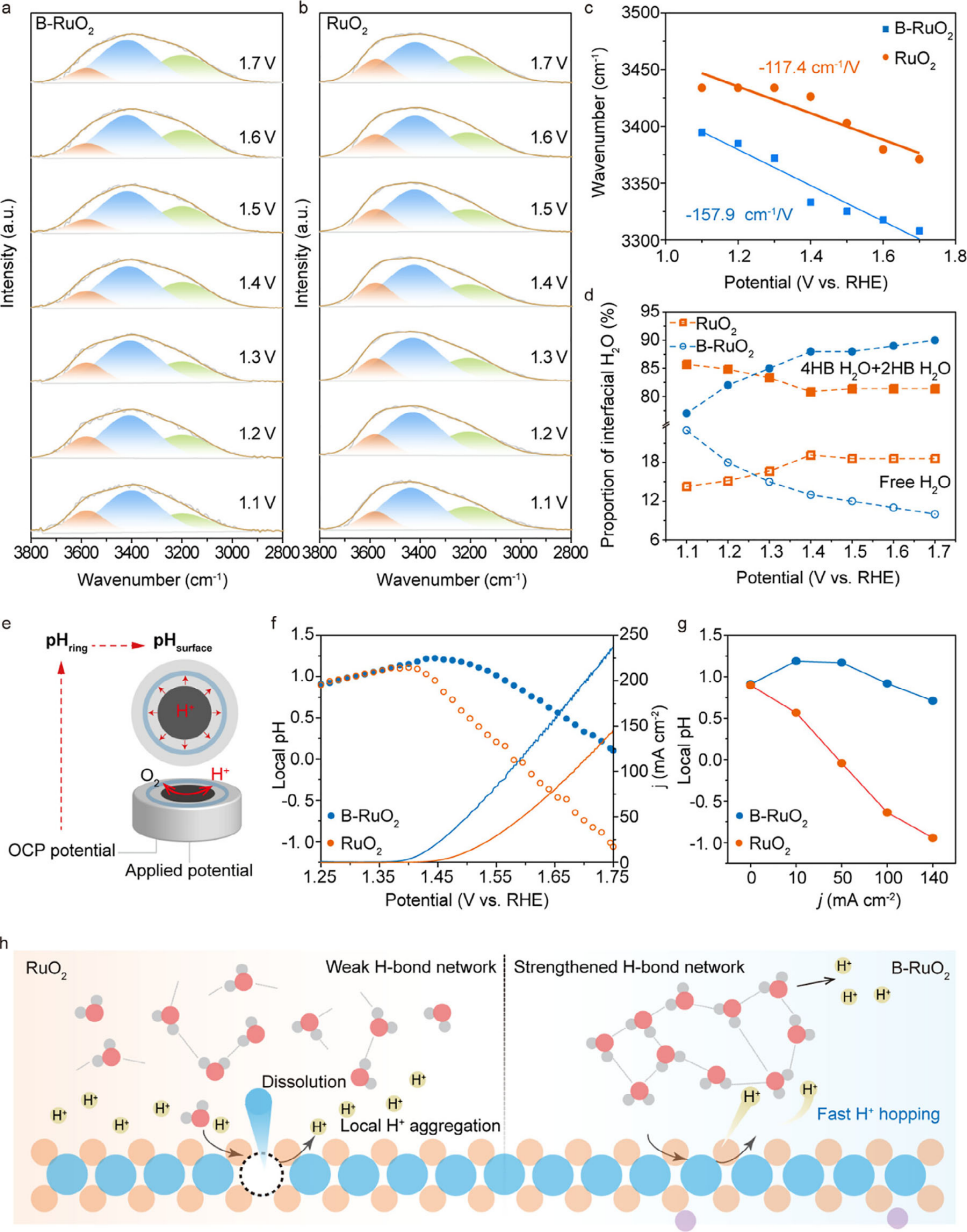
Interfacial H_2_O structure and local pH. In situ ATR‐SEIRAS of interfacial water for (a) B‐RuO_2_ and (b) RuO_2_. (c) Changes of the O─H stretching wavenumber (ν_O–H_) of interfacial water on B‐RuO_2_ and RuO_2_ electrodes with the electrode potential. (d) The proportions of 4HB‐H_2_O, 2HB‐H_2_O, and free H_2_O at varied potentials of B‐RuO_2_ and RuO_2_. (e) The schematic illustration of RRDE for monitoring local pH. (f) Changes of local pH on B‐RuO_2_ and RuO_2_ electrodes with the electrode potential. (g) Changes in local pH on B‐RuO_2_ and RuO_2_ electrodes with the current densities. (h) Schematic diagram showing the structural collapse caused by the accumulation of protons on the surface of RuO_2_ and strong hydrogen bond network connectivity for rapid proton transport at the B‐RuO_2_ interface. Reproduced with permission from Ref. [56], Copyright 2025, American Chemical Society.

Furthermore, in situ ATR‐FTIR studies have corroborated pH‐dependent variations in water configurations [55, 56]. During OER, the O─H stretching band of H_2_O was deconvolved into three Gaussian peaks corresponding to four‐hydrogen‐bonded water (4HB‐H_2_O), two‐hydrogen‐bonded water (2HB‐H_2_O), and the dangling O─H bonds (free H_2_O) of the interfacial water at ∼3205, 3402, and 3575 cm^−1^, respectively. Figure 5 shows a redshift of the vibration peaks with the increased potential. The potential‐dependent proportion of the three peaks illustrates that the rise of 4HB‐H_2_O and 2HB‐H_2_O can facilitate rapid proton hopping, as evidenced by the reduced interfacial pH drop [56]. Ni et al. revealed that 4HB‐H_2_O predominated under strongly acidic conditions, whereas the proportion of 2HB‐H_2_O increased substantially under near‐neutral conditions [55].

It should be noted that under certain conditions—such as strongly hydrogen‑bonded environments (e.g., ice‑like water at interfaces), confined interfaces (e.g., water in nanochannels or between hydrophilic surfaces), or high electrochemical polarization—a fourth vibrational feature may emerge in the O─H stretching region. For example, under anodic polarization, a sharp peak at ∼3585–3612 cm^−1^ appears, assigned to non‑hydrogen‑bonded or isolated water molecules [23, 57, 58]. In strongly hydrogen‑bonded or confined environments, additional low‑frequency components (e.g., near ∼3200 cm^−1^ or lower) can arise from ice‑like or nanoconfined water structures, as recently reviewed by Yuan et al. [59]. However, for the majority of electrocatalytic interfaces discussed in this review—where water configurational changes are primarily driven by the applied potential and the EDL—the three‑component model (ice‑like, liquid‑like, and free water) provides a robust and widely adopted description.

### 3.2 Raman Spectroscopy

**FIGURE 6:**
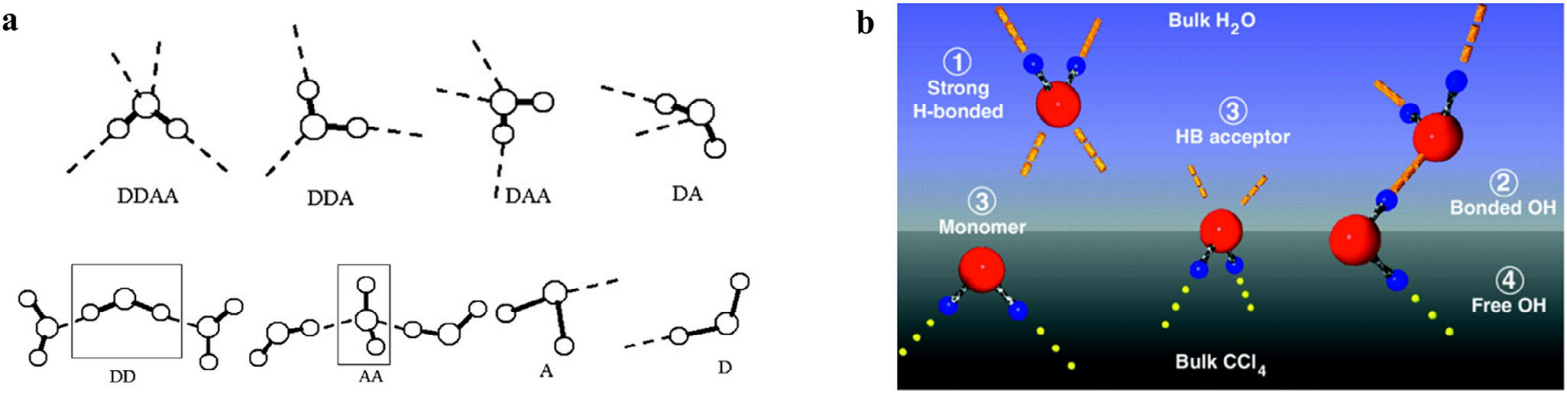
(a) Local hydrogen bond network of water molecules. Dashed lines represent hydrogen bonds. Under ambient temperature and pressure conditions, DDAA, DDA, DAA, and DA are the main hydrogen bond motifs in liquid water, while structures such as DD, AA, D, and A are negligible. Reproduced with permission from Ref. [60], Copyright 2009, Elsevier B.V. (b) Schematic depiction of water molecules at the CCl_4_/H_2_O interface probed by SFG. The dashed and dotted lines represent intermolecular interactions: H_2_O‐H_2_O and H_2_O‐CCl_4_, respectively. Reproduced with permission from Ref. [61], Copyright 2001, American Association for the Advancement of Science.

Depending on whether the hydrogen bonds formed by water molecules with adjacent species function as proton donors, proton acceptors, or a combination of both, local hydrogen‐bonded networks can adopt eight characteristic configurations: double‐donor double‐acceptor (DDAA), double‐donor single‐acceptor (DDA), single‐donor double‐acceptor (DAA), single‐donor single‐acceptor (DA), double‐donor (DD), double‐acceptor (AA), single‐donor (D), and single‐acceptor (A), as schematically depicted in Figure 6a. Under ambient temperature conditions, the predominant local hydrogen bond motifs are generally recognized to be DDAA, DDA, DAA, and DA. This observation highlights that, beyond the continuous three‐dimensional hydrogen‐bond network, liquid water also encompasses discrete cyclic or chain‐like substructures. As a direct spectral manifestation of this structural heterogeneity, the O‐H Raman stretching vibration region of liquid water consists of multiple characteristic bands corresponding to DDAA‐OH, DDA‐OH, DAA‐OH, DA‐OH, and free OH groups. The specific positions of these Raman peaks measured under standard conditions (275 or 290 K and 0.1 MPa) are summarized in Table 1 for reference [60].

**TABLE 1:** Fitting Results of O─H Stretching Raman Spectra of Water at 275 and 290 K under 0.1 MPa Pressure. Reproduced with permission from Ref. [60], Copyright 2009, Elsevier B.V.

Mode	275 K			290 K		
	Frequency	FWHM	Intensity (%)	Frequency	FWHM	Intensity (%)
DAA‐OH	3004	145	1.1	3014	130	0.8
DDAA‐OH	3227	228	40.9	3226	219	38.6
DA‐OH	3431	219	48.6	3432	228	53.6
DDA‐OH	3565	144	5.6	3572	129	4.3
Free OH	3633	110	3.6	3636	96	2.7

As the electrode potential is polarized negatively, the interfacial water layer undergoes a structural reorganization from a parallel arrangement (Figure 2a) to an H‐down configuration (Figure 2c), which yields enhanced Raman scattering intensity due to its altered polarizability. Through in situ Raman spectroscopy, Jiang and co‐workers delineated three characteristic O─H stretching bands associated with distinct hydrogen‐bonding states: a peak at ∼3200 cm^−1^ corresponding to strongly bonded, tetrahedral water; a band near 3400 cm^−1^ assigned to moderately bonded water; and a feature at ∼3600 cm^−1^ attributed to weakly bonded or nearly free O─H groups [62], which are named as 4HB‐H_2_O, 2HB‐H_2_O and free H_2_O, respectively [63].

To address the weak Raman signals from many catalytic materials, the groups of Sun and Li pioneered in situ electrochemical SHINERS [12]. This technique employs a silica shell that not only prevents nanoparticle aggregation but also effectively isolates the gold core from interfering with either the catalytic reaction or the Raman signal. The resulting spectra show a broad O─H stretching band between 3000 and 3600 cm^−1^, which can be deconvoluted into three characteristic components: a feature near 3600 cm^−1^ assigned to free water (dangling O─H bonds), a band around 3400 cm^−1^ corresponding to doubly‐coordinated hydrogen‐bonded “liquid‐like” water, and a peak at ∼3200 cm^−1^ attributed to tetrahedrally‐coordinated hydrogen‐bonded “ice‐like” water [12, 37].

### 3.3 Sum Frequency Generation Spectroscopy

SFG spectroscopy stands as a powerful and selective tool for probing the structure of water at interfaces [64]. As a second‐order nonlinear optical technique, SFG is inherently surface‐specific: its signal vanishes in centrosymmetric media such as bulk electrolytes or electrode interiors, thereby naturally isolating contributions from the top few molecular layers at the interface [65]. This contrasts sharply with conventional linear vibrational spectroscopies (e.g., IR and Raman), where signals are overwhelmingly dominated by the bulk phase [66]. Thus, a key advantage of SFG is its direct sensitivity to molecular orientation. Changes in the net dipole alignment of interfacial water molecules manifest as shifts in the phase angle between resonant and non‐resonant spectral contributions, providing a quantitative measure of orientational order [67]. Specifically, high‐quality SFG spectra are typically obtained using a dual‐tunable laser system in which two optical parametric oscillators (OPOs) are synchronously pumped by a mode‐locked Nd:YAG laser. This configuration enables precise tuning of the infrared frequency while maintaining high spectral resolution and signal‐to‐noise ratio, making it particularly well suited for in situ studies of potential‐dependent interfacial water restructuring [68].

The SFG spectra reveal characteristic O─H stretching bands that directly reflect the hydrogen‐bonding environment of interfacial water. The peak near 3600 cm^−1^ is attributed to non‐hydrogen‐bonded (“free” O─H) species [29, 61, 69]. The feature around 3400 cm^−1^ arises from weakly hydrogen‐bonded O─H groups, corresponding to the asymmetric stretching mode (ν_3_) of disordered water molecules, which is often associated with under‐coordinated, liquid‐like water structures [61, 68]. In contrast, the peak near 3200 cm^−1^ is assigned to the symmetric O─H stretching mode (ν_1_) of tetrahedrally coordinated water molecules, indicating strong hydrogen‐bonding characteristic of an ice‐like water network, as labeled ① in Figure 6b [61, 67]. The intensity ratio between these two major bands (∼3200 and ∼3400 cm^−1^) serves as a sensitive indicator of interfacial water disorder. Analysis of this ratio shows that at negative potentials, where hydrogen‐bonding is weaker, the interfacial water layer exhibits greater disorder, with water molecules predominantly adopting an H‐down orientation toward the electrode. As the potential shifts positively and approaches the PZC, the hydroxyl groups tend to align randomly or parallel to the electrode surface. When the potential becomes more positive than the PZC, the SFG signal intensity increases again, reflecting a reordering of the interfacial water structure under the applied field [61].

### 3.4 X‐ray Absorption Spectroscopy (XAS)

XAS is realized by capturing either incident photons or secondary electrons generated from the decay of core holes induced by absorbed X‐rays [70]. Endowed with inherent element specificity, XAS is capable of furnishing insights into the electronic structure surrounding excited atoms, and such information is highly sensitive to the local atomic arrangement and chemical microenvironment [71]. For the structural elucidation of water via XAS measurements, an electrochemical flow cell configuration is indispensable, which is equipped with an internal gold electrode and irradiated externally with synchrotron radiation X‐ray sources, with subsequent analysis focused on the absorption spectrum of soft x‐rays at the oxygen K‐edge. XAS encompasses two distinct detection modalities: the total fluorescence yield (TFY) mode, which features a micrometer‐scale penetration depth and thus exhibits bulk‐phase sensitivity; and the total electron yield (TEY) mode, which relies on the collection of secondary electrons. Owing to the short mean free path of electrons in condensed‐phase materials, the TEY mode probes the top 2–3 layers of water molecules, thus providing prominent surface sensitivity.

**FIGURE 7:**
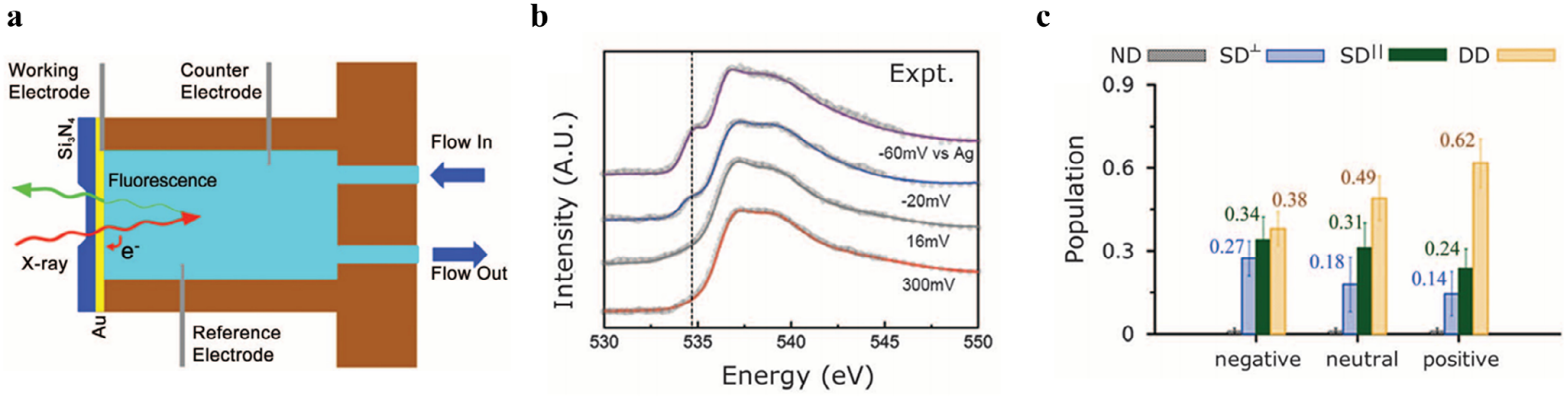
(a) Schematic diagram of the electrochemical cell for XAS measurements. (b) TEY O K‐edge XAS spectra of water molecules collected on the gold electrode surface under different potential conditions (relative to the silver reference electrode) (the experimentally determined PZC was 80 mV) (c) Relationship between the distribution of hydrogen‐bonded water molecules and the charge state of the Au surface, where ND denotes no hydrogen bond donor, SD^⊥^denotes one hydrogen bond donor with water molecules perpendicular to the surface. SD^||^ denotes one hydrogen bond donor with water molecules parallel to the surface, and DD denotes two hydrogen bond donors. Reproduced with permission from Ref. [36], Copyright 2014, American Association for the Advancement of Science.

XAS requires specialized cell designs to maintain electrochemical control while enabling X‐ray transmission. A representative flow cell, illustrated in Figure 7a, incorporates a thin Si_3_N_4_ membrane (∼100 nm) that seals the liquid electrolyte from the high‐vacuum beamline environment and includes integrated reference and counter electrodes for precise potential control. The sensitivity of XAS to the hydrogen‐bonding environment of water is demonstrated in Figure 7b, which compares experimental and theoretical O K‐edge spectra of the water/Au interface at varying potentials. The pre‐edge feature near 535 eV originates from under‐coordinated O─H groups (so‐called dangling H‐bonds), a characteristic signature of disordered, liquid‐like water that is notably absent in the highly ordered configuration shown in Figure 2a. The main edge at ∼537 eV corresponds to water molecules with partially saturated hydrogen bonds, while the post‐edge around 540 eV reflects molecules embedded in a fully coordinated, tetrahedral hydrogen‐bond network. Figure 7c reveals how the interfacial electric field directly tunes this hydrogen‐bonding landscape. Under negative bias, the field drives water reorientation with H atoms pointing toward the electrode, breaking interfacial H‐bonds and increasing the population of dangling O─H groups, thereby enhancing the pre‐edge intensity. Conversely, under positive bias, the interface becomes enriched in double‐donor water molecules that form two strong H‐bonds, leading to the near‐complete suppression of the pre‐edge signal. These potential‐dependent spectral shifts provide direct electronic‐structure evidence that the applied bias can switch the interfacial water layer between a H‐bond‐disrupted and a H‐bond‐enhanced state, with direct implications for associated electrocatalytic processes [36].

### 3.5 Electrochemical Impedance Spectroscopy (EIS)

**FIGURE 8:**
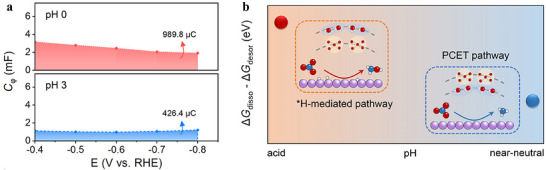
(a) Plots of Cφ vs. potential for Bi NSs in 0.5 M H_2_SO_4_ (pH 0) and 0.005 M H_2_SO_4_ (pH 3) electrolytes. (b) Schematic diagram showing the effects of the interfacial water structure on the selectivity of NH_2_OH. The purple, blue, red, and white spheres represent Bi, N, O, and H atoms, respectively. Reproduced with permission from Ref. [55], Copyright 2025, American Chemical Society.

EIS, an in situ technique, can reveal polarization processes during electrolysis and facilitate the analysis of electrochemical reaction dynamics [72, 73, 74]. The variation in water configurations is inextricably tied to H adsorption, with EIS as a pivotal and irreplaceable technique for interrogating H adsorption behaviors on electrode surfaces. In systematic investigations of the water electrolysis reaction, a hallmark of EIS Nyquist plots is the consistent emergence of two well‐defined semicircles, irrespective of whether the reaction medium is acidic or alkaline. Researchers have unambiguously assigned the high‐frequency semicircle in EIS profiles to the mass transfer process of adsorbed H_ad_ species, which directly reflects the kinetic rate of underpotential‐deposited hydrogen (H_upd_). Conversely, the low‐frequency semicircle corresponds to the charge transfer step, encoding the reaction dynamics of overpotential‐deposited hydrogen (H_opd_) [37]. For reactions involving the key intermediate adsorbed H species, EIS provides critical, otherwise inaccessible kinetic insights into the reaction mechanism. Ni et al. conducted EIS test to monitor the adsorbed H during NH_2_OH electrosynthesis at different pH [55]. The adsorption pseudocapacitance (*C*_φ_) can be integrated with potential to obtain the adsorption charge of ^*^H (Q_H_). As shown in Figure 8a, the catalyst exhibits a larger Q_H_ in the electrolyte with lower pH which promotes the hydrogenation of ^*^NO and the desorption of ^*^NH_2_OH. This process can be attributed to the dominant 4HB‐H_2_O in acid solution (Figure 8b) [55].

When synergistically integrated with complementary characterization techniques, EIS further enables a comprehensive understanding of interfacial water structure, addressing critical knowledge gaps that cannot be resolved through isolated analytical approaches. For example, Eckhard et al. proposed alternating‐current scanning electrochemical microscopy (AC‐SECM), which generally combines EIS with scanning electrochemical microscopy and successfully visualized the electrochemical process [75].

### 3.6 Multiscale Computational Simulation and Machine Learning

Computations based on first principles can aid in analyzing molecular‐scale water configuration changes [56, 76, 77]. Theoretical calculations can capture subtle variations in bond lengths and bond angles during proton transfer, thereby guiding the design of water configurations in catalytic reactions through ML [53, 78, 79, 80]. DFT calculation is widely used for analysis due to its balance of speed and accuracy, and has become an essential tool in heterogeneous surface catalysis for exploring reaction pathways and evaluating the catalytic capabilities of specific materials and active sites [81, 82]. They can predict the optimal adsorption configurations of surface species [83, 84, 85]. Therefore, computing the adsorption configurations of interfacial water can reveal the adsorption state of water on surfaces in reactions where water serves as a reactant, thereby validating experimental results [86]. Previous studies have shown that surface doping can induce asymmetric adsorption of water structures, altering O–H bond energies and accelerating water dissociation [78].

Very recently, Chen et al. provided a comprehensive multiscale theoretical framework for water‐solid interfaces, bridging atomic‐scale DFT/AIMD calculations, mesoscale MD simulations of hydrogen‐bond networks and nanoconfinement effects, and macroscopic external field modulation. Their work articulates how theoretical simulations can overcome key experimental limitations—capturing transient ion configurations, rapid proton transfer events, and short‐lived intermediates—thereby offering a predictive foundation for rational interfacial design [87].

**FIGURE 9:**
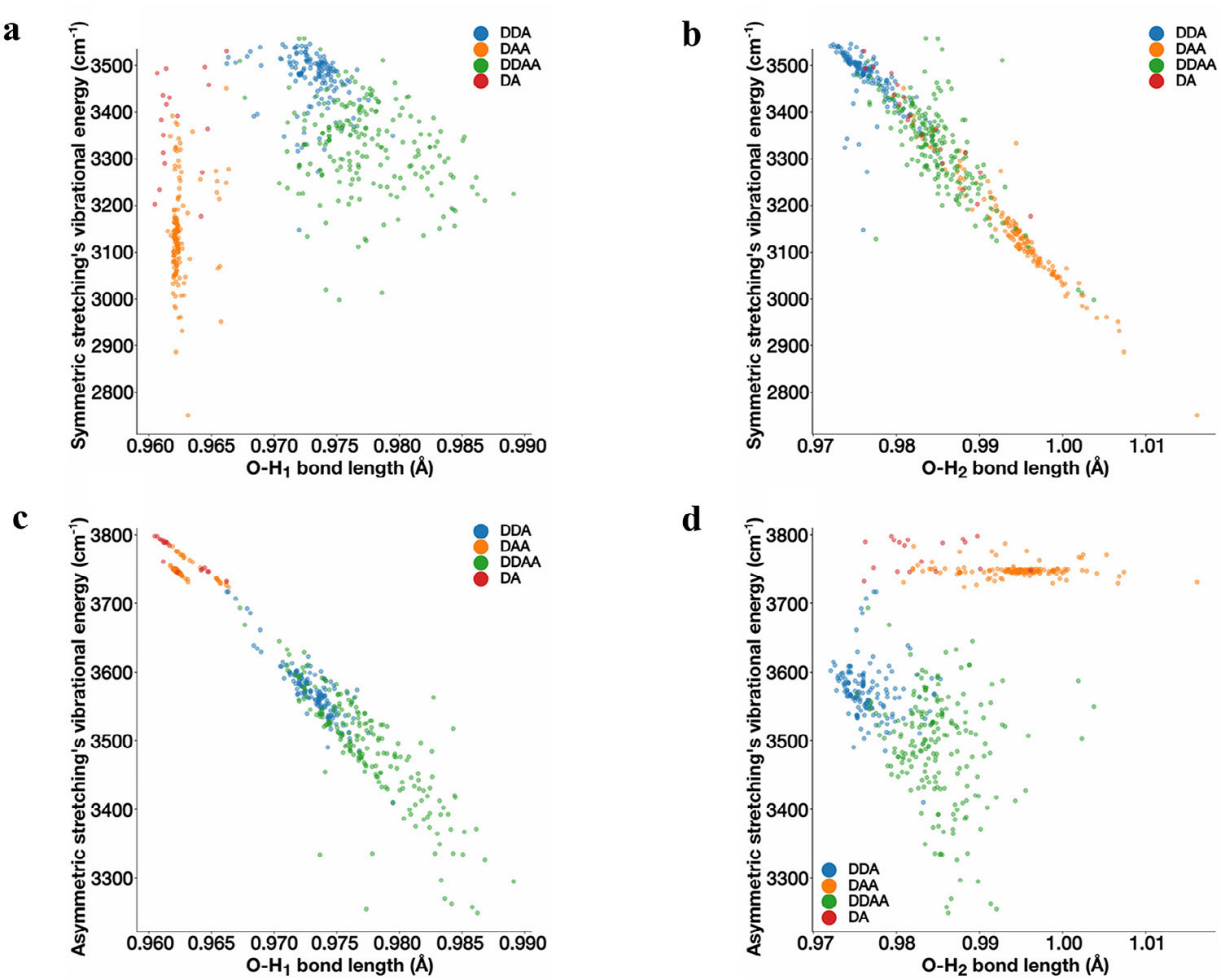
Bond lengths plotted as a function of (a) and (b) symmetric stretching's vibrational energy and (c) and (d) asymmetric stretching's vibrational energy. Reproduced with permission from Ref. [53]. Copyright 2025, PCCP Owner Societies.

ML has emerged as a powerful tool in catalyst design and mechanistic studies. By integrating experimental and theoretical data, ML can rapidly identify key descriptors governing catalytic performance, optimize catalyst compositions, and accelerate the discovery and synthesis of high‐performance electrocatalysts [88]. Ang et al. employed a ML approach to construct a neural network model to systematically analyze the relationship between O─H stretching vibrational frequencies and the structure of the hydrogen‐bonding network in water clusters of various sizes based on multi‐scale computational simulations [53]. Due to the sensitivity of the O─H stretching vibration to hydrogen bond strength, where stronger hydrogen bonds lead to a redshift in frequency, and weaker hydrogen bonds cause a blueshift, it can serve as a probe for hydrogen bonding. As shown in Figure 9, the study found that donor hydrogen bonds have a significantly greater impact on vibrational energy than acceptor hydrogen bonds. For symmetric stretching modes, the frequencies follow the order from low to high: DAA < DDAA < DDA. While for asymmetric stretching modes, the order is DDAA < DDA < DAA. This difference arises because the symmetric stretching mode is primarily controlled by the strength of the donor hydrogen bond associated with the longer O─H bond (O─H_2_), whereas the asymmetric stretching mode, in structures without free H atoms (DDA, DDAA), is governed by the donor hydrogen bond strength of the shorter O─H bond (O─H_1_), while in DAA, the stretching of the free H atom dominates.

Despite the widespread use of DFT, AIMD, classical MD, and ML potentials in studying interfacial water, each method has intrinsic limitations that must be carefully considered [89]. Most DFT calculations for interfacial water are performed at constant charge, whereas electrochemical interfaces operate at constant potential. This discrepancy can lead to incorrect predictions of potential‐dependent water orientation and surface charging [89]. Then, the explicit vs. implicit solvent trade‐off constitutes a core challenge: fully explicit solvent models preserve detailed structural information about ion double layers and diffusion behaviors but are fundamentally limited by electronic structure calculation overhead to tens to hundreds of picoseconds and several hundred atoms in system size. Conversely, implicit solvent treatments lose critical structural information regarding ion double‐layer formation and diffusion dynamics [87, 90]. Ion distributions in computational setups often lack physical realism. When studying pristine H_3_O^+^ or OH^−^ charge defects in pure water without additional electrolyte ions, the absence of screening counterions means the computational voltage does not represent local voltage drops near metal electrodes, lacking the double‐layer capacitance essential for modeling realistic electrochemical cells. Such setups consequently fail to establish well‐defined relationships between electrode potential and surface charge density required for reliable predictions [90]. He et al. systematically demonstrated that AIMD simulations are typically limited to sub‐nanosecond timescales and a few hundred atoms, leading to poor statistics for diffusional properties, while finite‐size effects critically compromise simulation reliability [91]. Besides, the cost of AIMD remains prohibitive despite algorithmic advances. Although combining ML with path integral contraction schemes has achieved two orders‐of‐magnitude acceleration, this approach still cannot eliminate the fundamental cost barrier [91]. Moreover, nuclear quantum effects (NQEs) due to hydrogen's low mass significantly influence hydrogen bond network structure and dynamics—zero‐point energy and quantum tunneling alter vibrational density of states and symmetry broadening in hydronium and hydroxide systems [92].

Finally, validation of machine‐learning potentials (MLPs) emerges as a growing concern: MLPs trained on DFT data inherit DFT's systematic errors, raising extrapolation stability questions when facing new chemical environments. While active learning and enhanced sampling strategies improve training data diversity, maintaining generalization capability and error control under new chemical conditions remains an open question. Consequently, cautious interpretation of computational results coupled closely with advanced experimental characterization techniques remains essential for reliable conclusions about interfacial water behavior.

### 3.7 Summary of the Techniques to Evaluate Interfacial Water

In summary, the techniques discussed in this section offer complementary windows into interfacial water. Vibrational spectroscopies (ATR‑SEIRAS, Raman, SFG) directly probe hydrogen‑bonding environments and molecular orientation, with ATR‑SEIRAS providing high surface sensitivity, SHINERS enabling single‑crystal studies, and SFG offering intrinsic surface specificity. XAS delivers element‑specific electronic structure information but requires synchrotron access. EIS provides indirect kinetic correlation without direct structural resolution. On the theoretical side, DFT offers atomic‑scale mechanistic insight but suffers from constant‑charge approximations and high computational cost; classical MD enables large‑scale, long‑time simulations but relies on force fields that may fail at interfaces; AIMD delivers ab initio accuracy but is limited to small systems and short timescales; and MLPs bridge the accuracy‑efficiency gap, yet their reliability depends critically on training data quality and transferability. No single technique can fully capture the complexity of interfacial water. A multi‑technique, multi‑scale strategy—combining vibrational spectroscopy for structural information, electrochemical methods for kinetics, and theoretical simulations for mechanistic interpretation—is essential for a holistic understanding of interfacial water in electrocatalysis.

## 4 Evolution of Interfacial Water Configurations Across Different Reactions

Under an applied bias, the hydrogen bond network structure of interfacial water within the EDL exerts a significant influence on the electrochemical performance of electrode materials [29, 70]. As such, the deliberate manipulation of interfacial water has emerged as a robust and versatile strategy for boosting electrochemical performance. To date, a broad range of catalytic reactions have been reported to be impacted by interfacial water configurations, including: HER, OER, HOR, CO_2_RR, carbon monoxide reduction reaction (CORR), ECH, and the electrochemical synthesis of NH_2_OH, among other key processes [27, 62, 93, 94, 95, 96].

### 4.1 Hydrogen Evolution Reaction and Hydrogen Oxidation Reaction

The water electrolysis process involves complex multi‐step pathways that require precise coordination of reactant delivery, product removal, and charge transfer across solid‐liquid‐gas interfaces [97]. For the HER, early studies used changes in the adsorption energy of the intermediate adsorbed H species as an activity descriptor, yet this descriptor proved insufficient to explain phenomena observed in alkaline media [98, 99, 100, 101]. Given the significant differences between HER in alkaline and acidic environments [102], Koper and colleagues at Leiden University proposed a pH‐dependent PZC. Using a kinetic model for the hydrogen adsorption step, they found that the hydrogen adsorption barrier depends on how far the electrode potential lies from the PZC. They attributed the influence of PZC on the activation barrier for hydrogen adsorption to the restructuring of interfacial water, which accommodates charge transfer across the EDL. As pH increases, the PZC shifts away from the HER/HOR potential, reducing the degrees of freedom of interfacial water and requiring higher energy to facilitate charge transfer through the double layer [32]. This results in a higher reorganization energy for OH^−^ transport in the double‐layer region [84]. Consequently, the PZC reflects the extent of interaction between water and the interfacial field, ultimately governing the kinetics of HER/HOR [28].

**FIGURE 10:**
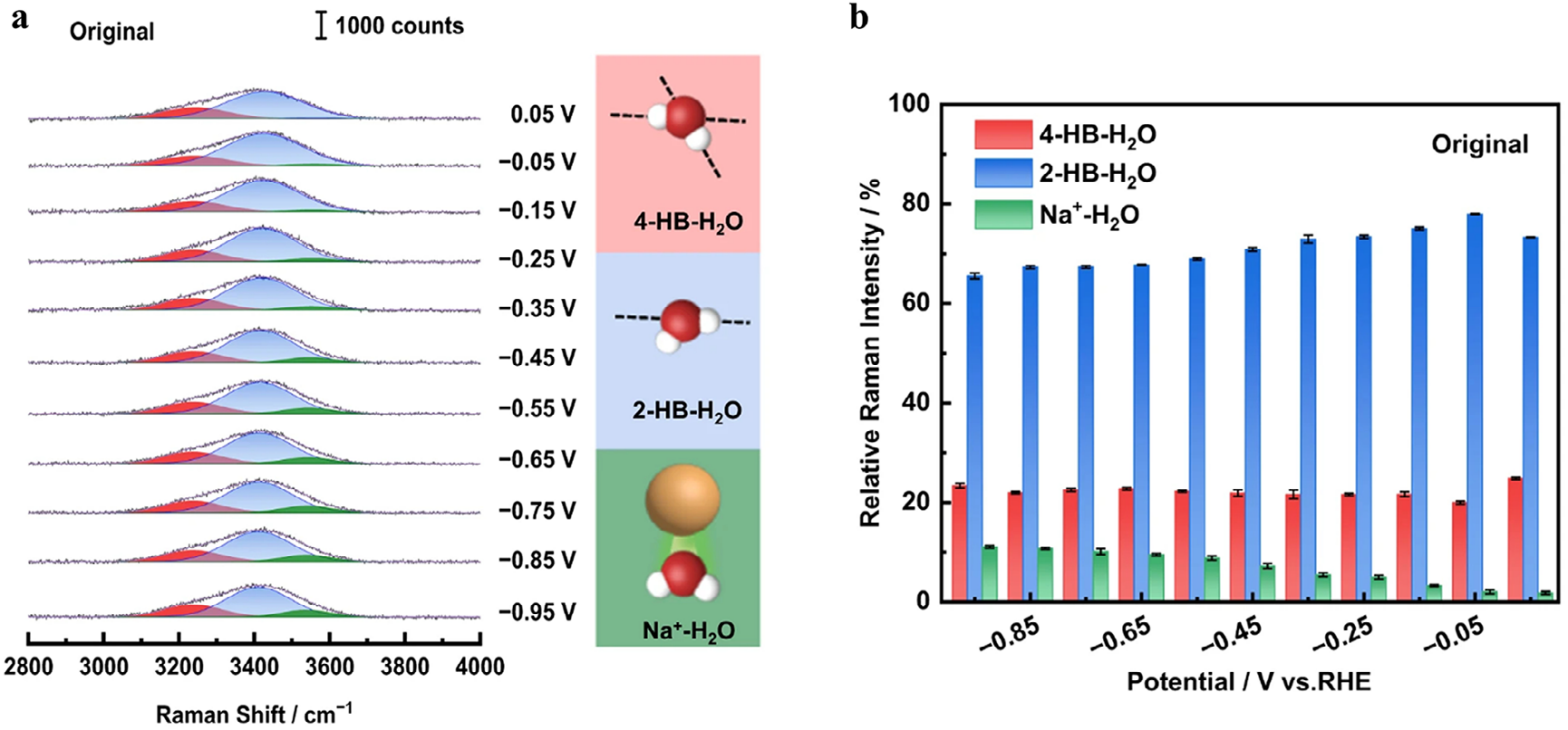
(a) In situ Raman spectra of interfacial water on the Ru surface. The Gaussian fittings of the three O─H stretching modes (ν_O‐H_) corresponding to 4HB‐H_2_O, 2HB‐H_2_O, and Na^+^‐H_2_O are shown in red, blue, and green, respectively. (b) Normalized Raman intensities of ν_O‐H_ at the HER potential. Reproduced with permission from Ref. [104], Copyright 2023, Springer Nature.

Xu and coworkers verified through surface‐enhanced infrared spectroscopy that the pH‐dependent H adsorption on Pt originates from the structure of interfacial water [103]. The water structure, which is affected by pH and the structure of proton donors, has emerged as a descriptor for the HER reaction [18]. Under the electrocatalytic HER potential, the electric field induces the orientational reconstruction of water molecules and disrupts the hydrogen bond network. If the interfacial water is prone to reorganization, charge transfer across the EDL will be rapid. Conversely, if the interfacial water is rigid and difficult to reorganize, charge transfer through the double layer will be sluggish [27]. Sun and coworkers also proposed using interfacial water structure as a descriptor for the HER. With increasing pH, the structure of the first water layer shifts from acting as a proton acceptor (H atoms pointing toward the electrode surface) to a proton donor (O atoms pointing toward the electrode surface). In alkaline media, this structural reorganization modifies the reactivity of interfacial water, following the trend in water dissociation activation energy: dangling O–H bonds < low‐coordination water < tetrahedrally coordinated water [37]. They further suggested that optimizing the adsorption of H and OH intermediates can orient the H atoms of interfacial water molecules toward the electrode surface, thereby enhancing HER kinetics [37]. In parallel, the Li group utilized in situ electrochemically enhanced Raman spectroscopy to monitor dynamic changes in water configurations under HER conditions. Their measurements revealed a pronounced increase in the population of 2HB‐H_2_O near the onset potential for hydrogen evolution. Upon further cathodic polarization, the spectral intensity of 2HB‐H_2_O decreased substantially, accompanied by a marked rise in signal from Na^+^‐H_2_O. This spectral evolution suggests a progressive conversion from 2HB‐H_2_O to Na^+^‐H_2_O during the HER process (Figure 10) [104].

Interfacial water plays a decisive role in HER/HOR. Recent advances have shown that the proportion of free water or weakly hydrogen‐bonded water (e.g., 2HB‐H_2_O, Na^+^‐H_2_O) correlates positively with HER/HOR activity, as these configurations lower the O─H bond dissociation barrier. Cation‐hydrated water structures (e.g., Na^+^‐H_2_O) have been identified as particularly active species, with their population increasing under cathodic polarization. Interfacial water studies are especially important for HER/HOR because the reaction mechanism shifts from H_3_O^+^ reduction in acid to H_2_O dissociation in base; understanding this shift requires molecular‐level insight into how water molecules are activated at the electrode surface. Future efforts should focus on designing catalyst surfaces that promote the formation of free or weakly hydrogen‐bonded water at HER potentials.

### 4.2 Oxygen Evolution Reaction

**FIGURE 11:**
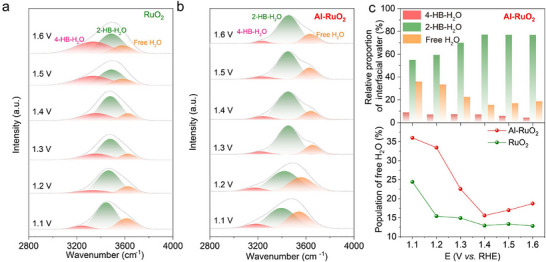
Interfacial Water and OER Reaction Kinetics. (a) Analysis of OH stretching vibration of interfacial water on RuO_2_ (b) Analysis of OH stretching vibration of interfacial water on Al‐RuO_2_ (c) Relative proportions of 4HB‐H_2_O, 2HB‐H_2_O and free water on Al‐RuO_2_ (top); comparison of free water proportions on RuO_2_ and Al‐RuO_2_ (bottom). Reproduced with permission from Ref. [69], Copyright 2025, American Chemical Society.

During the electrocatalytic OER process, typically hydrophilic surfaces can enhance water adsorption and dissociation, thus accelerating reaction kinetics [105, 106]. Meanwhile, the arrangement of reconstructed interfacial water induced by differences in surface properties can boost the local concentration of reactants and proton transfer capability [69]. By doping Al into RuO_2_ catalysts, studies have revealed that the Al‐RuO_2_ surface exhibits a gradual increase in doubly hydrogen‐bonded water (2HB‐H_2_O) with rising potential (Figure 11a,b), and its proportion of free water is significantly higher than that of pure RuO_2_ (Figure 11c). The authors attributed the greater abundance of free water on the Al‐RuO_2_ surface to its higher propensity for generating OH species, which can capture free water via hydrogen bonding, creating an interfacial microenvironment favorable for OER. Additionally, Al acts as a Lewis acid site, engaging in acid‐base interactions with water molecules to further enhance the surface's ability to trap free water. The improved OER performance resulting from more free water stems from an increased concentration of reactive species and a strengthened hydrogen‐bonding network that promotes proton‐transfer efficiency. This shifts the reaction pathway from the lattice oxygen mechanism (LOM) to the adsorbate evolution mechanism (AEM), thereby lowering the overall reaction barrier [69].

The Oener team investigated how interfacial solvation modulates OER transition states, proposing an “entropy‐enthalpy compensation” phenomenon that highlights the critical role of electric‐field‐induced ordering of interfacial water molecules in governing reaction kinetics. Their study revealed that at high overpotentials, excess charge accumulates at the catalyst–solution interface, generating a strong local electric field that reorients and reorganizes water molecules. This increases the proportion of O‐down oriented water until saturation is approached. From a kinetic perspective, the alignment of disordered water molecules is an entropy‐reducing process that tends to slow the reaction. However, the restructured water network simultaneously creates a microenvironment that promotes reactant orientation and activation while accelerating proton transfer, which manifests kinetically as an enthalpy gain. The resulting trade‐off between entropy loss and enthalpy gain leads to an entropy‐enthalpy compensation effect, ultimately enhancing the overall reaction rate. Notably, when the potential reaches a transition point, SHG measurements indicate that the ordering of interfacial water molecules saturates, implying that the pre‐organization of the water network is complete. Beyond this point, the reaction becomes kinetically controlled, with additional overpotential primarily used to stabilize reaction intermediates and lower the activation barrier. Importantly, this transition potential correlates with phase changes in the catalyst, underscoring the strong coupling between the surface charge state of the catalyst and the configurations of interfacial water molecules [107].

In OER, interfacial water serves as both the oxygen source and the proton donor, and its configuration directly affects the deprotonation steps (^*^OH→^*^O→^*^OOH) and O─O bond formation. Recent advances have demonstrated that increasing the proportion of free water at the interface enhances OER activity by facilitating electron transfer across the double layer and lowering the water dissociation barrier. Interfacial water studies are particularly important for OER because the reaction involves multiple proton‐coupled electron transfer steps, and the efficiency of deprotonation is governed by the hydrogen‐bond network connectivity at the interface. Understanding how to tune the interfacial water structure—from 4HB‐H_2_O to free H_2_O—offers a new dimension for catalyst design beyond conventional electronic structure optimization.

### 4.3 Carbon Dioxide Reduction Reaction

**FIGURE 12:**
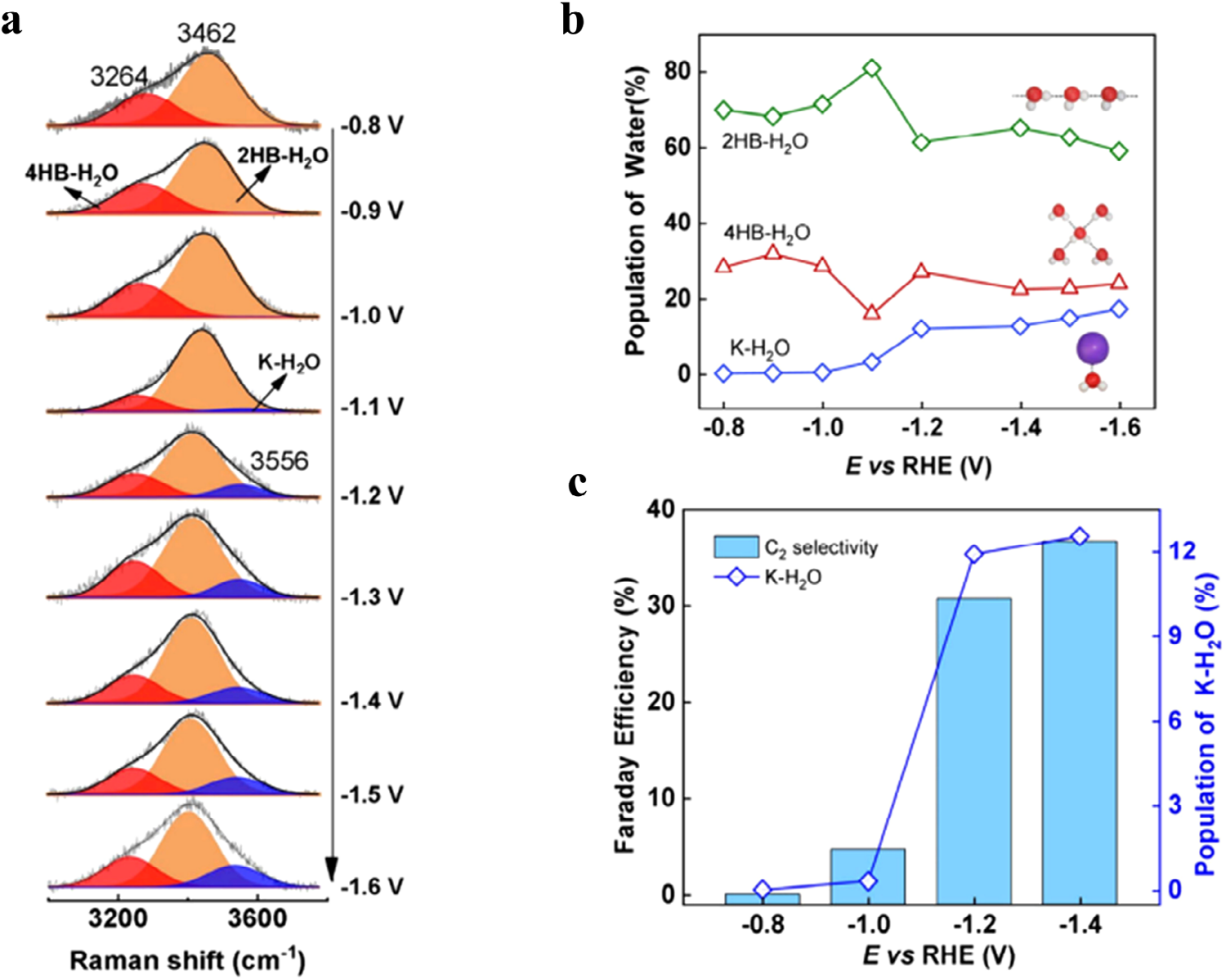
In situ Raman spectra of interfacial water on Cu (100) surface (a) in 0.1 M KHCO_3_ solution saturated with CO_2_. The Gaussian fitting curves of interfacial water are denoted by red (4HB‐H_2_O), orange (2HB‐H_2_O), and blue‐violet (K^+^‐H_2_O), respectively. (b) Variation of the content proportions of 4HB‐H_2_O, 2HB‐H_2_O, and K^+^‐H_2_O at the interfacial configuration region. (c) Correlation between K^+^‐H_2_O content and the selectivity of C_2_ product. Reproduced with permission from Ref. [31], Copyright 2025, American Chemical Society.

**FIGURE 13:**
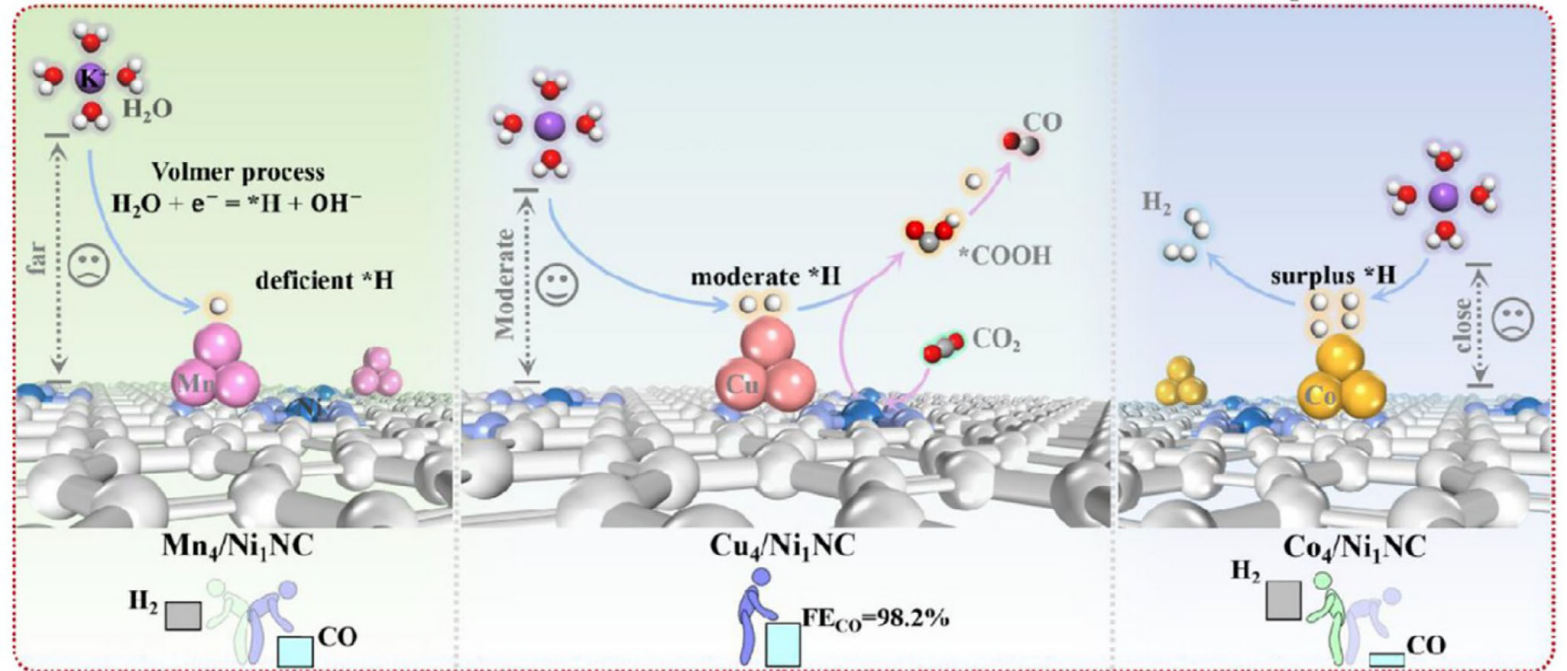
Schematic diagram of electrocatalytic CO_2_RR on the M_4_/Ni_1_NC surface. Reproduced with permission from Ref. [95], Copyright 2025, Wiley‐VCH GmbH.

Interfacial water exhibits dual functions in the electrocatalytic CO_2_RR: on the one hand, it acts as a proton donor, supplying protons to reaction intermediates and thus facilitating hydrogenation; on the other hand, interfacial water competes with reaction intermediates for active sites, altering the progression of side reactions [30, 108]. The research team led by Li found that, as illustrated in Figure 12, with the decrease of potential, the proportions of 4HB‐H_2_O and 2HB‐H_2_O decrease significantly, while the content of K^+^‐H_2_O increases remarkably. Moreover, in the presence of K^+^, interfacial water adopts a configuration with one hydrogen atom pointing downward, which makes it difficult to form hydrogen bonds with ^*^CO. This hinders the hydrogenation of ^*^CO to COH and renders C─C coupling the dominant reaction pathway [31]. Similarly, the Yu research team constructed a dual‐site tandem catalyst (M_4_/Ni_1_NC) to systematically investigate how different metal clusters (M_4_) influence interfacial water structure and thereby regulate ^*^H supply and CO_2_ reduction performance. Using in situ FTIR to analyze interfacial water configurations, they found that the proportion of K^+^‐H_2_O varies across different catalysts. This proportion reflects the strength of interaction between K^+^ ions and water molecules in the interfacial region and correlates with the trend in the Stark slope, indicating that Co_4_ and Cu_4_ clusters facilitate closer proximity of K^+^ ions to the surface, enhancing water ordering. Moreover, the K^+^‐H_2_O ratio exhibits a volcano‐shaped relationship with CO Faraday efficiency—a moderate proportion supplies sufficient adsorbed hydrogen for CO_2_ hydrogenation without triggering excessive HER, which aligns with computational simulations. This study establishes a quantitative link between the K^+^‐H_2_O ratio in interfacial water and electrochemical CO_2_ reduction performance, revealing the critical role of interfacial water configurations in the CO_2_RR (Figure 13) [95].

**FIGURE 14:**
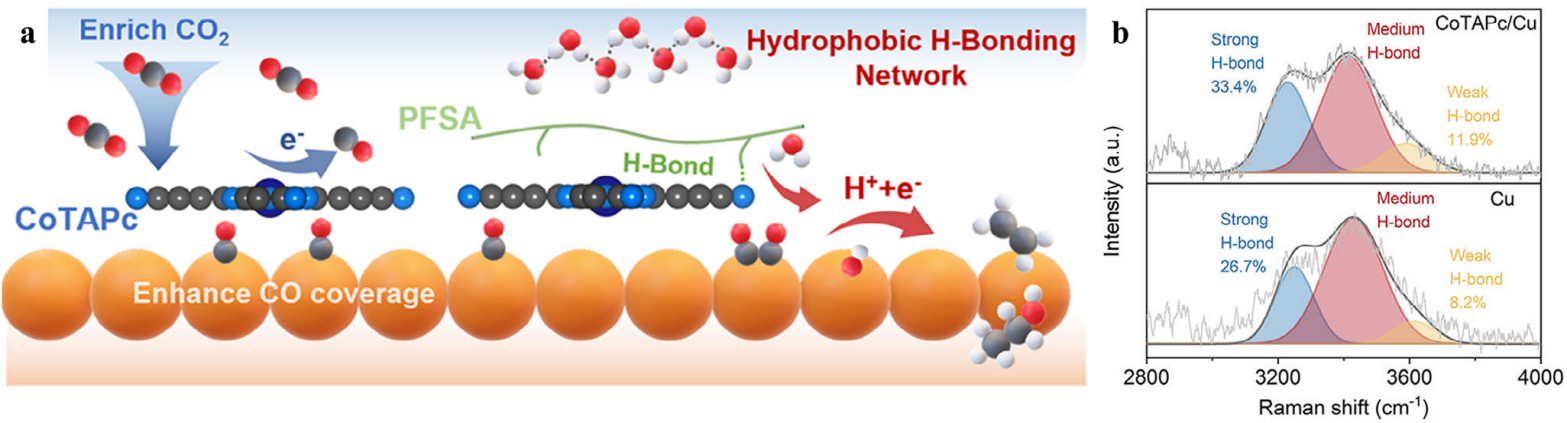
(a) Schematic of the CoTAPc/Cu catalyst design and its interfacial regulation strategy. (b) Comparison of Raman spectra for CoTAPc/Cu and pure Cu at −1.26 V (vs. RHE), highlighting O─H stretching bands from adsorbed water molecules. Reproduced with permission from Ref. [62], Copyright 2025, Wiley‐VCH GmbH.

For the electrocatalytic CO_2_RR to C_2_ products, the Jiang group, on the other hand, utilized the hydrogen bonding network formed by co‐assembly of CoTAPc and PFSA to induce the reorganization of interfacial water molecules from a disordered state into spatially confined water clusters (Figure 14a), thereby achieving directional proton transport. The Raman spectroscopy results in Figure 14b show that, compared with the Cu electrode, the amount of water molecules with weak hydrogen bonds on the CoTAPc/Cu electrode increases significantly, indicating weakened interactions between water molecules and thus easier dissociation. Meanwhile, the content of water molecules with strong hydrogen bonds rises to a certain extent, indicating the presence of a spatially confined hydrogen‐bond network. This network facilitates the directional transfer of protons to ^*^CO intermediates and promotes C─C coupling. In addition, the hydrophobic interface restricts non‐selective proton diffusion, thereby effectively suppressing the HER [62].

Interfacial water plays a dual role in CO_2_RR: it supplies protons for hydrogenation steps but also competes with CO_2_RR via the HER. Recent advances have revealed that cation‐hydrated water (e.g., K^+^‐H_2_O) and weakly hydrogen‐bonded water promote C─C coupling and C_2_ product selectivity by hindering the hydrogenation of ^*^CO to ^*^COH. Conversely, 4HB‐H_2_O can suppress HER by increasing the water dissociation barrier. Interfacial water studies are especially important for CO_2_RR because selectivity—the central challenge of this reaction—is directly governed by the competition between ^*^CO hydrogenation (leading to CH_4_) and C─C coupling (leading to C_2_ products), and this competition is modulated by the local water structure and cation hydration. Tailoring the interfacial water environment may therefore offer a powerful strategy for steering product distribution.

### 4.4 Carbon Monoxide Reduction Reaction

**FIGURE 15:**
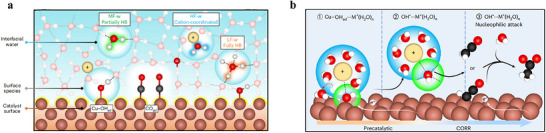
(a) The local microenvironment of Cu during alkaline CORR. Low‐frequency water (orange) corresponds to fully hydrogen‐bonded water (four H‐bonds), medium‐frequency water (green) to partially hydrogen‐bonded water (two to three H‐bonds), and high‐frequency water (blue) to cation‐coordinated water. (b) Proposed non‐covalent interaction mechanism for acetate formation. Hypothesis for the high acetate production mechanism in alkaline CORR. In brief, during the pre‐catalytic stage, the non‐covalent interactions between adsorbed hydroxyl groups (Cu─OH_ad_) and interfacial water molecules enhance the medium‐frequency water. Upon desorption of Cu─OH_ad_···M^+^(H_2_O), locally generated OH^−^···M^+^(H_2_O)_n_ complexes‐distinct from free OH^−^ in the bulk solution‐reside within the EDL on the Cu catalyst surface, thereby promoting the selective conversion of CORR to acetate. Reproduced with permission from Ref. [109], Copyright 2025, Springer Nature.

The structural dynamics of interfacial water during alkaline CORR are governed by non‐covalent interactions with adsorbed OH on Cu, which in turn dictate product selectivity (Figure 15a). Operando spectroscopic studies reveal a potential‐dependent evolution: as the potential shifts from −0.5 to −1.1 V, medium‐frequency water (partially H‐bonded, ∼3400 cm^−1^) increases markedly, high‐frequency water (cation‐coordinated, ∼3600 cm^−1^) decreases, while low‐frequency water (fully H‐bonded, ∼3200 cm^−1^) remains stable. This trend is ascribed to OH‐induced restructuring of the interfacial hydration layer. This behavior is explained by a proposed Cu–OH_ad_–M^+^(H_2_O)_n_ non‐covalent complex. Interaction within this complex leads to partial dehydration, forming a coordinated OH‐M^+^ adduct. Upon OH desorption, the resulting OH^−^–M^+^(H_2_O)_n_ species resides within the double layer, stabilizing the local water structure and accounting for the rise in medium‐frequency water. Notably, these OH^−^–M^+^(H_2_O)_n_ clusters also act as nucleophiles that attack the ^*^HC═C═O intermediate, steering the reaction pathway toward acetate formation (Figure 15b) [109].

In aprotic media, the formation of water microclusters has been shown to dramatically alter CORR selectivity. Fejzić et al. demonstrated that in acetonitrile‐water mixtures supporting water‐water clustering, significant amounts of methane and ethylene (up to 22% FE for C_2_H_4_) are produced, whereas microhomogeneous mixtures (e.g., DMSO‐water) yield only HER. The key descriptor is the hydrogen‐bond donation ability (Kamlet–Taft α parameter) of water: higher α correlates with more hydrogenated CORR products [110]. Furthermore, Liu et al. showed that by lowering the local concentration of K^+^ via an anion‐exchange ionomer—which increases water activity at the electrified interface—the selectivity can be steered from acetate toward ethanol, achieving an ethanol FE of 42.5% and an ethanol/acetate ratio increase of 3.7‐fold at 700 mA cm^−2^ [111].

Interfacial water studies are particularly important for CORR because product distribution—acetate vs. ethanol/ethylene—is dictated by the local water structure and the presence of cation‐hydroxide adducts, which are distinct from species in bulk solution. Unlike CO_2_RR, where CO is an intermediate, CORR starts directly from CO, making water structure effects more direct and more readily deconvoluted. Understanding how water clustering, hydrogen‐bond donation ability, and cation‐water interactions steer CORR pathways opens new opportunities for electrolyte design and for tuning selectivity.

### 4.5 Electrocatalytic Hydrogenation Reaction

**FIGURE 16:**
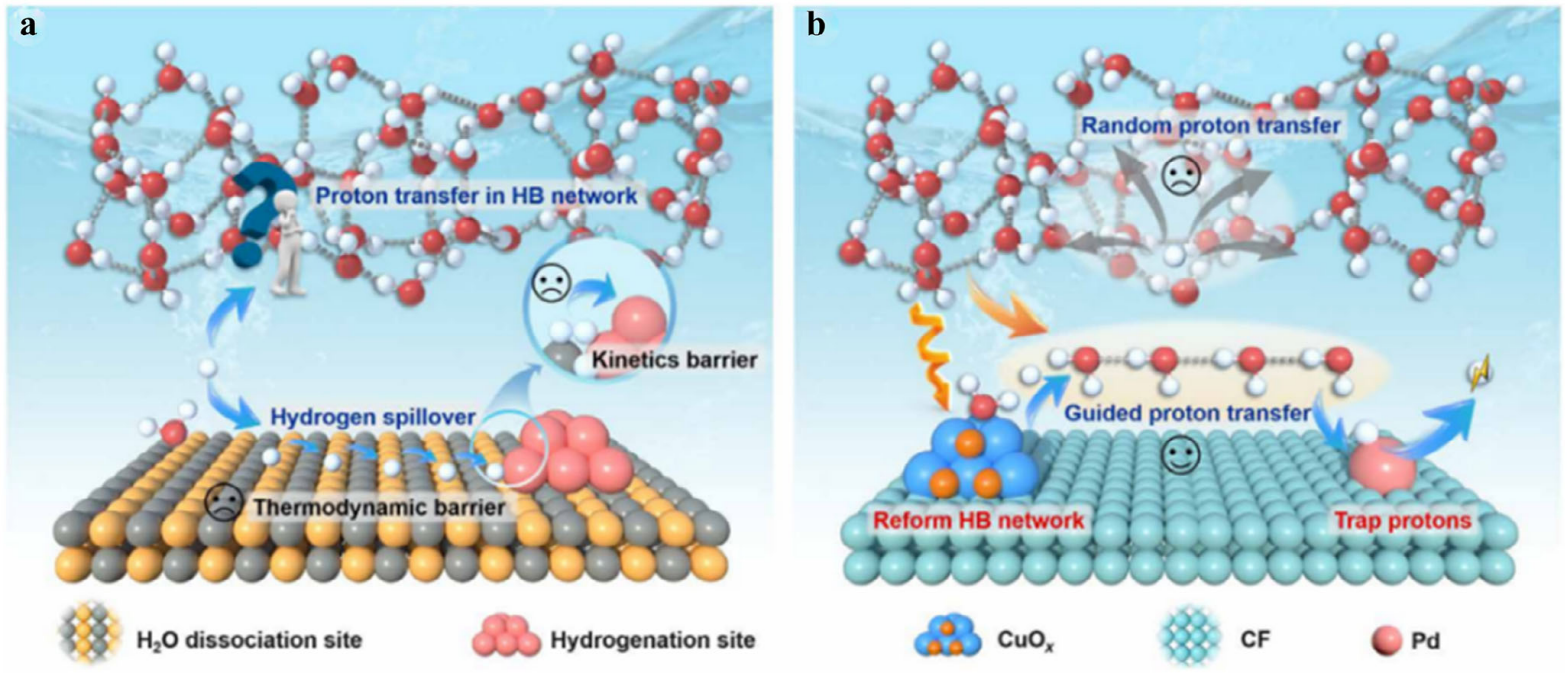
Schematic illustration of hydrogen transfer pathways. (a) Traditional hydrogen spillover vs. potential interfacial hydrogen‐bond network‐mediated proton transfer. (b) Random proton transfer in the intrinsic interfacial hydrogen‐bond network vs. directional proton transfer along the restructured interfacial hydrogen‐bond network. Reproduced with permission from Ref. [63], Copyright 2025, American Association for the Advancement of Science.

In ECH reactions, water serves as the sole hydrogen source, and interfacial water restructuring directly governs proton transport efficiency and selectivity against HER [63]. Studies have shown that on unmodified Pd_1_–CF electrode surfaces, interfacial water molecules predominantly exist as 4HB‐H_2_O, forming a three‐dimensional hydrogen‐bond network. While this structure stabilizes water, it results in random and inefficient proton transport pathways (Figure 16a). Researchers addressed this by introducing CuO_x_ to induce water restructuring and direct proton transfer. The hydrophilic CuO_x_ forms strong interactions with water, weakening water‐water hydrogen bonds and promoting the transformation of 4HB‐H_2_O into 2HB‐H_2_O. Due to the higher flexibility of the 2HB‐H_2_O hydrogen‐bond network, it provides linear, low‐energy‐barrier channels for proton transport. Experimental results show that as the potential decreases from 0–1.2 V vs. RHE, the proportion of 2HB‐H_2_O increases significantly from 35.3% to 67.3%. Raman data indicate that protons are transported along the restructured water chains via the Grotthuss mechanism within this network. Computational results further reveal that the energy barrier for this process is only 0.6 eV, much lower than the 1.28 eV required for the traditional surface hydrogen spillover pathway. Moreover, the negatively charged Pd_6_^−^ adsorbs H^+^ from water through electrostatic interactions, inducing a configuration where the hydrogen atoms of water point toward the catalyst surface. This configuration promotes O–H bond stretching, weakens the bond energy, facilitates bond cleavage, and ultimately releases protons. Therefore, the potential‐dependent evolution of water configurations is the key structural foundation for enhanced proton transport efficiency. Simultaneously, the directed proton transport pathway suppresses the competing hydrogen evolution side reaction, improving the selectivity of the hydrogenation reaction (Figure 16b) [63].

Beyond catalyst design, microenvironment modulation has also been shown to influence selectivity through alteration of water accessibility. For instance, quaternary ammonium surfactants create a hydrophobic interface that hinders water transfer, suppressing HER and improving alkene selectivity [112]. Thiolate‐modified Pd nanotips form anion‐hydrated cation networks that concentrate reactants and accelerate water electrolysis [113]. More broadly, Chadderton et al. distinguished ECH—which requires ^*^H generated via the Volmer reaction — from direct electroreduction, demonstrating that hydrogenation products arise from ECH pathways where water is the ultimate proton source [114].

Interfacial water studies are especially important for ECH because selectivity between desired hydrogenation and undesired HER is determined by the connectivity and orientation of the interfacial water network. Traditional thermochemical hydrogenation requires high‑pressure H_2_, while ECH using water as the hydrogen source offers a safer, more sustainable alternative. By directing proton transport along restructured 2HB‑H_2_O chains or creating hydrophobic microenvironments that control water accessibility, researchers can achieve high selectivity while maintaining activity—a capability that is unique to ECH. Interfacial water restructuring is critical for electrocatalytic hydrogenation, as it provides the proton source and governs proton transport efficiency. Recent advances have shown that transforming the interfacial water network from 4HB‐H_2_O to 2HB‐H_2_O via hydrophilic modifiers enhances proton transport through the Grotthuss mechanism while suppressing the competing HER. Notably, however, direct experimental evidence linking specific interfacial water configurations (e.g., 4HB‑H_2_O vs. 2HB‑H_2_O) to ECH performance remains scarce, and this represents an important direction for future research in this field.

### 4.6 Electrochemical Synthesis of Hydroxylamine

**FIGURE 17:**
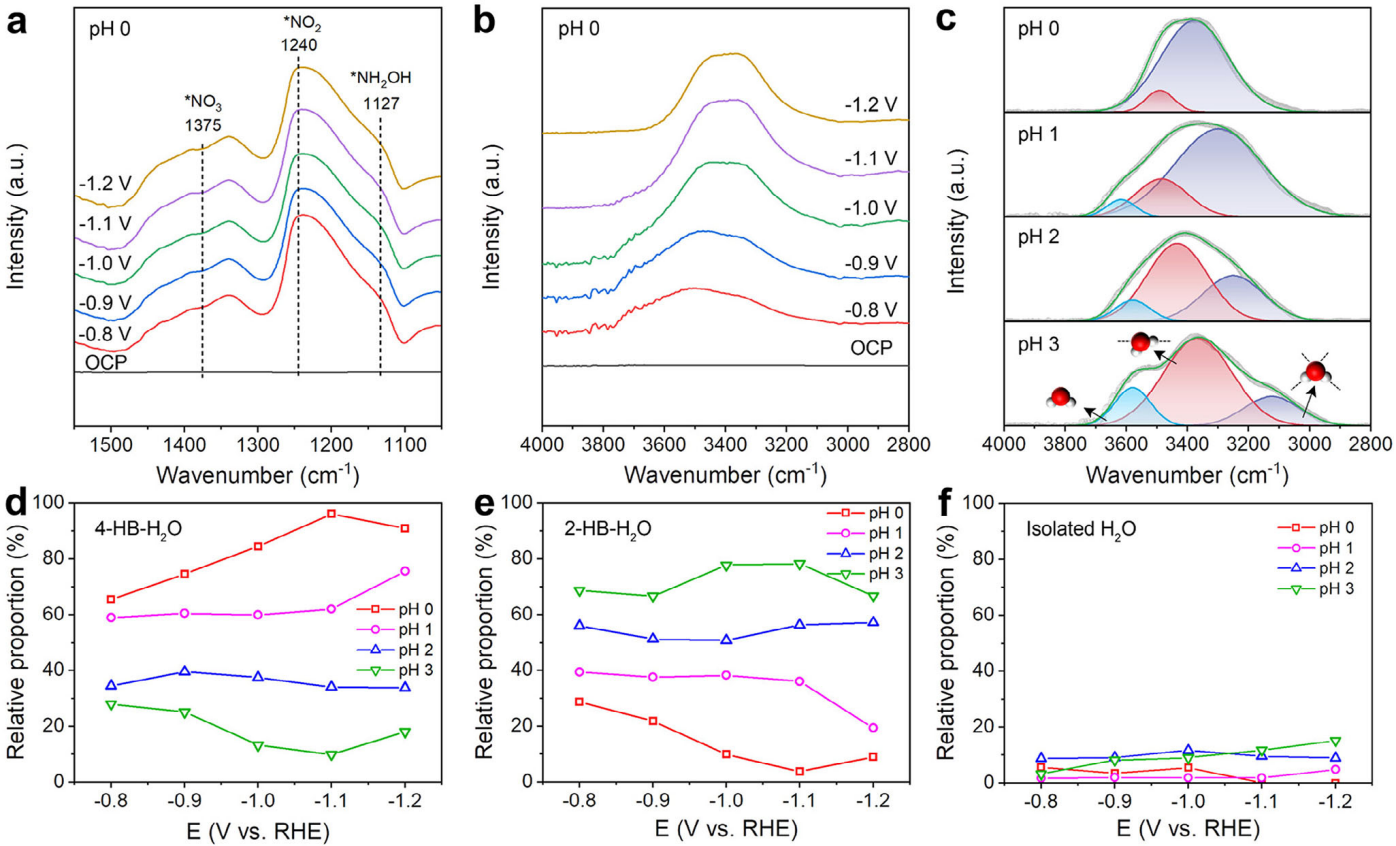
(a) In situ ATR‐FTIR spectra for Bi NSs in an electrolyte at pH 0. (b) O─H stretching bands in the in situ ATR‐FTIR spectra for Bi NSs in the electrolyte of pH 0. (c) O─H stretching bands in the in situ ATR‐FTIR spectra at −1.2 V vs RHE under different pHs. The relative proportions of (d) 4‐HB‐H_2_O, (e) 2‐HB‐H_2_O, and (f) isolated H_2_O. Reproduced with permission from Ref. [55], Copyright 2025, American Chemical Society.

The configurations of interfacial water critically govern reaction pathways in the electrochemical synthesis of NH_2_OH [55]. Ni et al. discovered that in strongly acidic electrolytes, interfacial water predominantly organizes into a 4HB‐H_2_O network, which creates ordered, low‐resistance proton‐transfer channels. According to the EIS and ATR‐FTIR (Figure 17), researchers attributed the promoted water dissociation to this strongly hydrogen‐bonded structure, thereby increasing the local concentration of adsorbed hydrogen (^*^H). Conversely, in near‐neutral media, the interfacial layer shifts toward a 2HB‐H_2_O configuration with weaker hydrogen bonding. This structural transition directly modulates proton availability and transport efficiency. Electron paramagnetic resonance studies further confirm that ^*^H serves as a key intermediate, as nitrate ions competitively consume surface ^*^H. Mechanistically, the 4HB‐H_2_O‐dominated interface in acidic media facilitates efficient ^*^H generation and transport, favoring the hydrogenation of NO to ^*^NHO with a low thermodynamic barrier (ΔG = 0.21 eV). Moreover, ^*^H weakens the adsorption of ^*^NH_2_OH on the Bi surface, preventing over‐reduction to NH_3_ and enhancing NH_2_OH selectivity. This water‐configuration‐dependent regulation of ^*^H supply and stabilization of intermediates highlights the decisive role of interfacial aqueous structure in steering reaction selectivity (Figure 17) [55].

Recent work introduced an interfacial water frustration strategy to achieve highly selective NO_3_^−^‑to‑NH_2_OH semireduction. By engineering the EDL through alkali metal cation modulation, the activation of interfacial H_2_O is precisely regulated to inhibit excessive ^*^H generation. Among the cations tested, K^+^ uniquely maintains an optimal equilibrium between ^*^H supply and demand. Raman spectroscopy reveals that as the cation radius increases from Li^+^ to Cs^+^, the population of 2HB‑H_2_O increases (from 77.9% to 83.8%), indicating progressive disruption of the hydrogen‑bond network, which frustrates water dissociation and reduces ^*^H supply [115].

In NH_2_OH electrosynthesis, the interfacial water configuration governs both the supply of ^*^H for NO hydrogenation and the stabilization of the ^*^NH_2_OH intermediate to prevent over‑reduction to NH_3_. In NH_2_OH electrosynthesis, the interfacial water configuration governs the supply of ^*^H for NO hydrogenation and prevents over‐reduction to NH_3_. Unlike HER or OER, NH_2_OH synthesis requires precise kinetic matching between ^*^H supply and demand, and the water frustration strategy provides a generalizable approach for achieving this matching.

### 4.7 Summary: The Central Functional Roles of Interfacial Water in Electrocatalysis

It is important to note that there is no universally “optimal” interfacial water configuration. Rather, the beneficial water structure depends on the specific kinetic requirements of each reaction. However, we can summarize that across the diverse electrocatalytic reactions discussed above—including HER, OER, CO_2_RR, CORR, ECH, and NH_2_OH synthesis—interfacial water consistently plays three central and interrelated functional roles.

First, it acts as a proton donor/acceptor and charge‐transfer mediator. In HER, water dissociation supplies adsorbed hydrogen (^*^H); in CO_2_RR and NH_2_OH synthesis, water provides protons for hydrogenation steps; in OER, water is the ultimate oxygen source. Second, the hydrogen‐bond network of interfacial water dictates proton transport efficiency via the Grotthuss mechanism. A more connected network (e.g., 4HB‐H_2_O) generally facilitates faster proton hopping, whereas disrupted networks (e.g., 2HB‐H_2_O or free water) lower the barrier for water dissociation. Third, interfacial water modulates reaction selectivity by competing with reactants for active sites or by stabilizing key intermediates. For example, a disordered or cation‐coordinated water layer suppresses undesired hydrogen evolution and promotes C─C coupling in CO_2_RR, while an ordered 4HB‐H_2_O network favors NH_2_OH formation over over‐reduction to ammonia. These condition‐dependent roles are summarized in Table 2 and discussed in detail in the respective subsections.

**TABLE 2:** Condition‐dependent roles of Interfacial Water in Electrocatalysis.

Reaction	Facilitate conditions	Beneficial water structure	Primary function
HER	Potential‐dependent; pH‐dependent; Electrolyte‐dependent	Free H_2_O	Fast water dissociation (Volmer step: H_2_O+e^−^→H^*^+OH^−^)
OER	Potential‐dependent; Catalyst‐dependent	Free water; 2HB‐H_2_O	Efficient deprotonation of ^*^OH to ^*^O and O─O bond formation
CO_2_RR	Potential‐dependent; Electrolyte‐dependent; Catalyst‐dependent	K^+^‐H_2_O	Selective hydrogenation of ^*^CO to C_2_ products while suppressing HER
CORR	Potential‐dependent	2HB‐H_2_O	Selective conversion of CO to acetate while suppressing HER and other side products.
ECH	Potential‐dependent; Catalyst‐dependent	2HB‐H_2_O	Efficient and directional proton transport for hydrogenation, while suppressing competing HER
NH_2_OH synthesis	pH‐dependent	4HB‐H_2_O	Abundant and continuous supply of adsorbed hydrogen (^*^H) for NO hydrogenation, while avoiding over‐reduction to NH_3_

In essence, while the specific consequences of water configuration vary with reaction type, the underlying regulatory mechanism is universal: the arrangement, hydrogen‐bonding state, and dynamic reorganization of interfacial water directly control the local proton activity, intermediate stabilization, and competitive adsorption at the electrified interface. Therefore, rationally tuning interfacial water configurations—rather than treating water as a passive solvent—represents a powerful and general strategy for optimizing electrocatalytic performance across a wide range of energy conversion reactions.

## 5 Methods for Regulating Water Configurations

### 5.1 Introduction of Local Force Fields

Hydrogen bonding among interfacial water molecules confers a stabilization energy of 20–40 kJ·mol^− 1^, biasing the system toward water‐water association rather than catalyst adhesion [116]. By introducing local hydrogen‑bonding interactions between surface hydroxyl groups and interfacial water molecules at the catalyst‑electrolyte interface, as demonstrated by Wen et al., this inherent preference can be disrupted. The field shifts the thermodynamic favorability toward water adsorption, steering molecular reorientation and enabling accelerated reaction progression [33].

**FIGURE 18:**
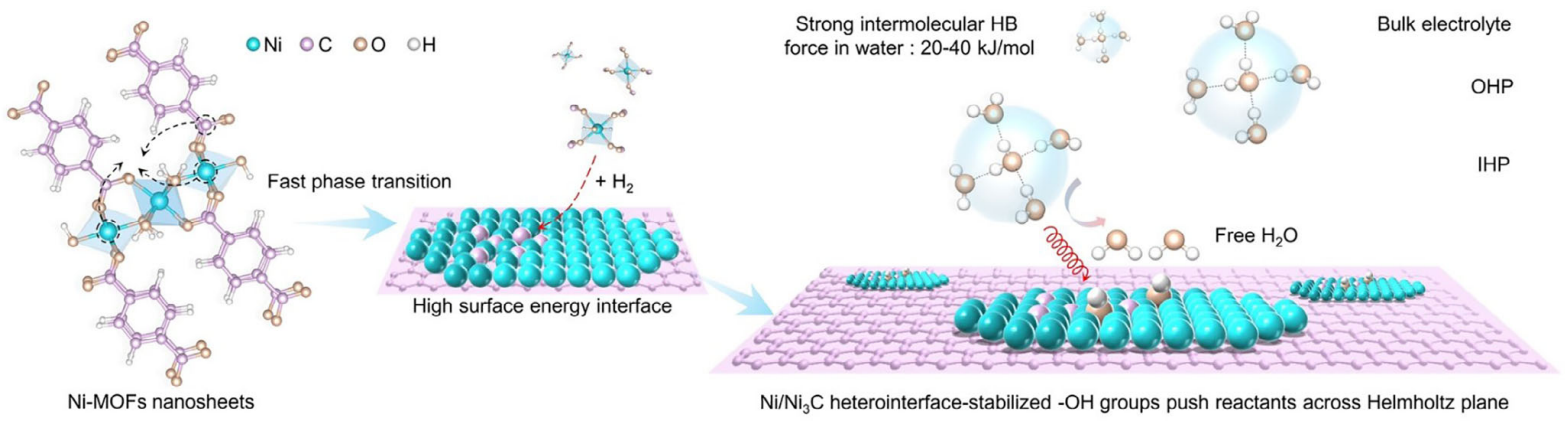
Schematic illustration of the OH─Ni/Ni_3_C heterostructure design, its atomic configuration, and the proposed water dissociation pathway. Reproduced with permission from Ref. [33], Copyright 2024, American Chemical Society.

Zhai and coworkers, using hydroxyl‐anchored Ni/Ni_3_C heterostructures as a prototype, introduced a local hydrogen‐bonding force field at the catalyst‐electrolyte interface, which can create a microenvironment rich in freely arranged H_2_O molecules near the IHP. As illustrated in Figure 18, during alkaline HER, hydroxyl groups exert a local force field on surrounding water molecules through hydrogen bonding, guiding their migration from the OHP toward the IHP. Using isotope‐labeled in situ Raman spectroscopy, the researchers observed that under the influence of hydroxyl groups, interfacial water undergoes a transformation from 4HB‐H_2_O to 2HB‐H_2_O and eventually to free‐water structures. The significant increase in the proportion of free water promotes the water dissociation step, while the accumulation of free water ensures a continuous supply of reactants under high‐current‐density conditions. DFT calculations revealed that the hydrogen‐bond interaction between hydroxyl groups and water has a strength of approximately 49 kJ·mol^− 1^, which is higher than that between bulk water molecules. Concurrently, hydroxyl‐induced electron redistribution enhances the electronegativity of oxygen atoms, facilitating hydrogen‐bond formation and water molecule polarization, thereby effectively lowering the water dissociation energy barrier [33].

### 5.2 Modulating the Electronic Structure

**FIGURE 19:**
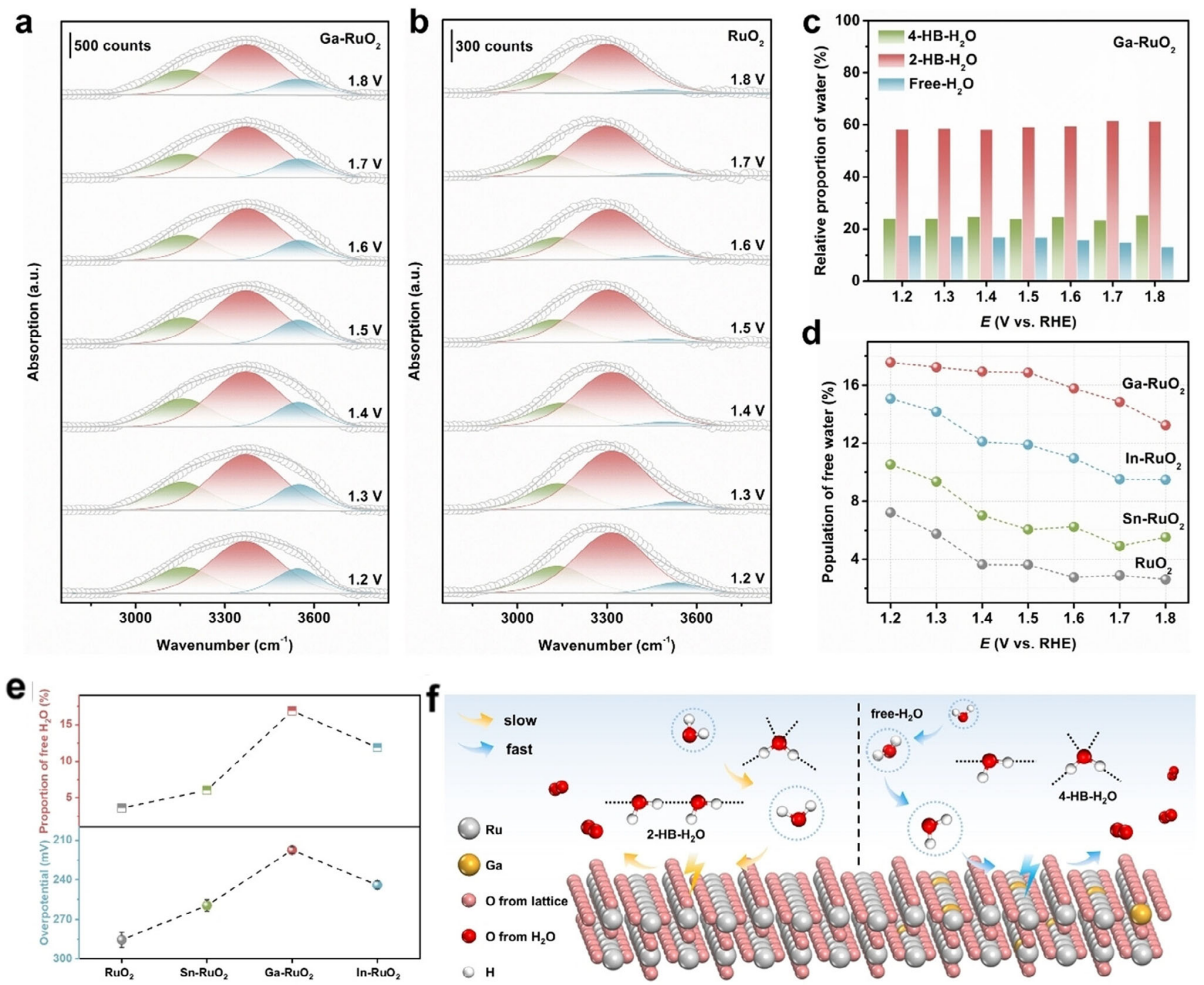
Potential‐dependent absorption intensities of interfacial water on (a) Ga−RuO_2_ and (b) RuO_2_ in 0.5 M H_2_SO_4_ measured by ATR‐SEIRAS on an Au electrode. (c) Proportions of different water types on the Ga−RuO_2_ catalyst. (d) Free‐water fractions on Ga−RuO_2_, In−RuO_2_, Sn−RuO_2_, and RuO_2_ at varying potentials. (e) OER overpotential at 10 mA cm^−2^ and free‐water ratio at 1.5 V for Ga−RuO_2_, In−RuO_2_, Sn−RuO_2_, and RuO_2_. (f) Schematic illustrating the dissociation of interfacial water on RuO_2_ and Ga−RuO_2_ surfaces. Reproduced with permission from Ref. [94], Copyright 2024, Wiley‐VCH GmbH.

Modulating the electronic structure offers an effective pathway to regulate the interfacial water configuration, which is largely governed by the catalyst's ligand environment and can be tailored through doping [117, 118, 119]. To systematically probe this effect on the acidic OER, Luo and colleagues designed a series of p‐block metal (Ga, In, Sn)‐doped RuO_2_ catalysts, focusing on the interplay among electronic structure, Ru─O covalency, and catalytic activity. As illustrated in Figure 19, theoretical calculations established the covalency trend: RuO_2_< Sn–RuO_2_< Ga–RuO_2_< In–RuO_2_. Electrochemically, this covalency exhibits a volcano‐type correlation with OER activity, with Ga‐RuO_2_ achieving the optimal performance. Operando ATR‐SEIRAS further revealed that the doped surfaces promote the dynamic transformation of interfacial water from 4HB‐H_2_O and 2HB‐H_2_O toward free water. This restructuring optimizes hydrogen‐bond network connectivity, creating a continuous, free‐water‐enriched interfacial region that facilitates electron transfer across the double layer, lowers the water dissociation barrier, and thereby enhances acidic OER kinetics [94].

**FIGURE 20:**
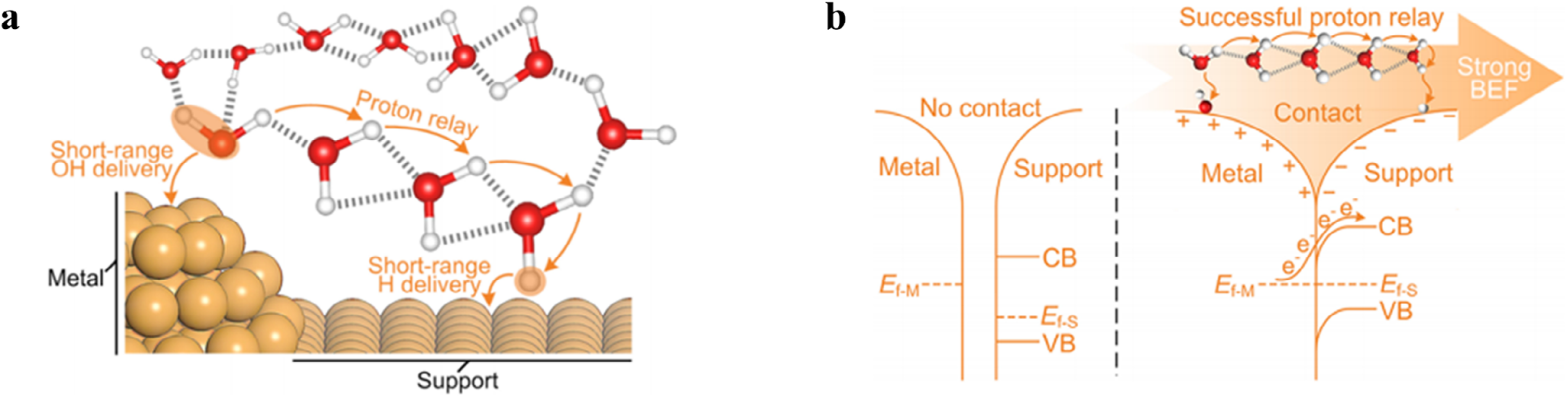
(a) Schematic of interfacial proton transfer via a hydrogen‐bond network enabling synergistic water dissociation between metal and support. (b) Schematic of the electronic structure and surrounding hydrogen‐bond network at a high‐work‐function metal‐support interface. (CB: conduction band, VB: valence band, E_f‐M_: metal Fermi level, E_f‐S_: support Fermi level). Reproduced with permission from Ref. [43], Copyright 2025, American Chemical Society.

However, some studies attribute the essence of modulating the electronic structure to optimizing water configurations on metal‐supported catalysts to a higher work function, which generates a strong built‐in electric field. This field optimizes the aqueous microenvironment within the EDL structure, thereby creating a synergistic mechanism for interfacial water dissociation [43]. As shown in Figure 20a, the Li research team proposed the transfer of OH from O‐down H_2_O to metal sites through a hydrogen‐bond network, along with proton transfer from O‐down H_2_O on the metal to H‐down H_2_O on the substrate. The authors supported Ru on CoP and CeCoP substrates. Ce, acting as a strong electron donor, induces significant electron transfer from Ce to neighboring Co and P upon doping. This enhances electron cloud density, raises the Fermi level, and lowers the work function (Figure 20b). By synthesizing model catalysts with varying atomic ratios to tune the work function, they observed that a lower work function correlates with poorer HER performance. This theoretically demonstrates that a high work function at the metal‐substrate interface promotes the formation of a hydrogen‐bond network around the catalyst via a built‐in electric field, establishing proton‐transfer channels [43].

### 5.3 Modulating the Interfacial Electric Fields

At the electrochemical interface, the surface charge density of the metal is modulated by the applied potential, thereby generating an interfacial electric field [15, 119, 120]. This electric field arises from charge separation between the metal electrode and the electrolyte solution and leads to a high concentration of free water at the IHP, thereby forcing the interfacial water molecules to adopt an “H‐down” orientation [15]. Duan and coworkers proposed that cations can regulate the adsorption of surface hydroxyl groups via this electric field, thereby optimizing the configurations of interfacial water molecules. Studies have found that moving from K^+^ to Na^+^ and then to Li^+^ enhances hydroxyl adsorption energy and increases hydroxyl coverage, which helps anchor hydrated cations and consequently alters the structure of the electrical double layer as well as the arrangement of interfacial water molecules. The strongest hydroxyl adsorption observed with Li^+^ is attributed to its highest charge density, which generates the most intense local electric field. Given the high polarity of adsorbed hydroxyl species (OH_ad_), they can act both as proton acceptors, attracting hydrogen atoms from nearby water molecules, and as proton donors to promote water dissociation. Computational simulations reveal that water molecules near OH_ad_ exhibit elongated and weakened O─H bonds, making them more susceptible to cleavage. Moreover, water molecules surrounding OH_ad_ form a stronger hydrogen‐bonding network, facilitating efficient proton transfer [121]. Other research suggests that Na^+^, due to its smaller size compared to K^+^, competes more effectively with H_3_O^+^ for surface adsorption sites, resulting in a higher overpotential for the HER. These findings collectively demonstrate that the electrolyte composition regulates interfacial water structure by influencing ion distribution and electric field profiles at the interface [122].

Further research indicates that the modulation of the interfacial electric field is reflected in shifts of the PZC [47]. In acidic media, the potential region for the HER/HOR lies closer to the PZC (around 0.34 V vs. RHE), where the reorganization energy required for protons to cross the EDL is relatively small. In contrast, under alkaline conditions, the PZC is located at approximately 1 V vs. RHE, and the larger water‐reorganization energy restricts OH^−^ transport across the EDL [28]. Using SFG spectroscopy, the Toney group observed four distinct O─H stretching vibration peaks whose intensities varied with applied potential. The peak at 3370 cm^−1^ corresponds to disordered liquid water with weak hydrogen bonding, predominantly located in the diffuse layer and showing minimal potential dependence. The peak at 3250 cm^−1^ is assigned to ice‐like water within the ion‐hydration layer. Its intensity is lowest near the PZC and increases with stronger interfacial fields, indicating enhanced ordering due to ion accumulation. The peak at 2970 cm^−1^ arises from specifically adsorbed water molecules interacting directly with the electrode surface via covalent or electrostatic forces, also exhibiting minimal intensity near the PZC, with its orientation modulated by the electric field. Finally, the peak at 2800 cm^−1^ is associated with H^+^ species, linked to negative surface charge accumulation and HER activity. It emerges near the PZC and intensifies at more negative potentials. This analysis reveals that under positive potentials, water molecules adsorb preferentially with oxygen oriented downward. Near the PZC, where the interfacial field is weakest, SFG signals are minimal, suggesting that water aligns parallel to the surface. At negative potentials, water undergoes reorientation with hydrogen atoms pointing downward, demonstrating clearly distinct potential‐dependent configurations [122]. Complementary thiol‐modification experiments further clarified these observations: the two peaks related to water directly interacting with the metal surface disappeared after thiol functionalization, while the two peaks associated with water in the diffuse layer remained. This confirms that the detected signals originate from the diffuse double layer and that the thiol layer screens the direct influence of the surface electric field on water molecules. These results validate that electric‐field regulation of water structure is mediated primarily through interactions between surface charge and interfacial water [122].

The Lum team regulates the interfacial electric field by modulating the nanocurvature of carbon supports, altering the arrangement, orientation, and configurations of water molecules at the electrode interface, thereby controlling the electrocatalytic CO_2_ reduction activity of single‐atom catalysts. Using SCN^−^ as a vibrational Stark effect probe, the authors measured the interfacial electric field strength via Raman peak shifts. Their findings revealed that the surface of carbon supports with high curvature generates a stronger local electric field, which enhances the enrichment of cations near the electrode and further affects the orientation of water molecules and hydrogen‐bond networks. Meanwhile, the electric field modulates the arrangement of water molecules by influencing the adsorption energies of dipolar intermediates such as COOH, CO, and ^*^OOH, thereby impacting the reaction rate and selectivity [4].

### 5.4 Modulating the Electrolyte Environment

**FIGURE 21:**
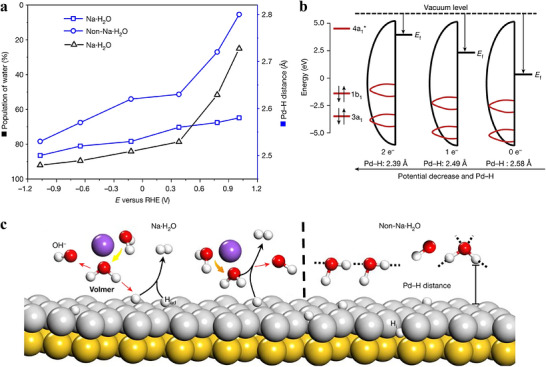
(a) AIMD simulations of Pd–H bond lengths in interfacial water for Na^+^‐H_2_O and non‐Na^+^‐H_2_O species (blue circles), along with the fraction of Na^+^‐H_2_O (black curve), under varying applied potentials. Na^+^‐H_2_O is defined as water molecules with an O─Na distance shorter than 3.2 Å. (b) Schematic illustration of the projected density of states (PDOS) for key orbital interactions between H_2_O (red line) and the underlying Pd atom (black line) in a Na^+^‐H_2_O cluster model as the potential decreases. (c) Schematic depiction of the water dissociation process at the interface on a Pd (111) surface supported on a gold monolayer–coated Au (111) substrate. Reproduced with permission from Ref. [12], Copyright 2025, Springer Nature.

Based on the hard‐soft acid‐base (HSAB) theory, hard Lewis acids, alkali metal cations (AM^+^), e.g., Li^+^, Na^+^, K^+^, Cs^+^, exhibit strong binding to hard Lewis bases such as OH^−^, but interact weakly with near‐neutral soft Lewis bases like OH_ad_ [123]. The binding energy resulting from the charge imbalance between OH^−^ and OH_ad_ facilitates the migration of OH species from the compact layer into the bulk electrolyte. As shown in Figure 21, Li and coworkers discovered that free water molecules form M^+^‐H_2_O coordination structures around cations. By comparing the Stark slopes of Na^+^‐H_2_O, 2HB‐H_2_O, and 4HB‐H_2_O, they found that Na^+^‐H_2_O is more sensitive to the local electric field than 2HB‐H_2_O and 4HB‐H_2_O, and tends to adopt an ordered configuration with hydrogen oriented downward more readily than hydrogen‐bonded water. Computational analyses reveal that the H atom in Na^+^‐H_2_O forms a shorter and stronger bond with surface Pd, thereby promoting water dissociation. Moreover, higher Na^+^ concentrations lead to an increased proportion of Na^+^‐H_2_O and a corresponding decrease in the HER overpotential.

Moreover, the valence of cations also significantly influences HER performance through multiple interrelated factors. Xu et al. systematically compared K^+^, Li^+^, and Ba^2+^ in alkaline HER and found that strongly hydrated cations (Ba^2+^) reduce the net orientation of interfacial water and its ability to reorient with the interfacial electric field, due to (i) rigid hydration shells that preserve centrosymmetric water coordination; (ii) effective charge screening that weakens the electric field felt by water molecules beyond the first hydration layer; and (iii) stronger water‐cation interactions that compete with water‐field alignment [124]. Rao et al. further showed that different cations alter the interfacial water hydrogen‐bonding network—Li^+^ promotes 4HB‐H_2_O while K^+^ promotes free H_2_O—which affects the activity of interfacial OH^−^ and the stabilization of oxygenated intermediates [125]. Huang et al. demonstrated that cations modify the interfacial static dielectric constant (Li^+^∼2.6, Cs^+^∼4.3), which directly impacts the reorganization energy of electron transfer and thus HER kinetics [126].

Importantly, the direction of cation effects (promotion vs. inhibition) is not universal but depends on the rate‐determining step. Bender et al. showed that on metals where water dissociation is rate‐limiting (Cu, Ag, Au), cations promote HER by stabilizing the transition state; whereas on metals where OH^−^ removal is rate‐limiting (Pt, Pd, Ir), cations inhibit HER [127]. Monteiro et al. further demonstrated that weakly hydrated cations can either promote or inhibit HER depending on their local concentration—low concentrations promote HER by stabilizing the water dissociation transition state, while high concentrations block active sites [128].

For divalent cations such as Ca^2+^, Sr^2+^, and Ba^2+^, these effects are amplified due to their higher charge density [12]. However, whether they enhance or inhibit HER depends critically on the specific reaction conditions—including the catalyst material, electrolyte pH, applied overpotential, and cation concentration—and should not be reduced to a single descriptor such as “ionic strength” or “cation valence”.

**FIGURE 22:**
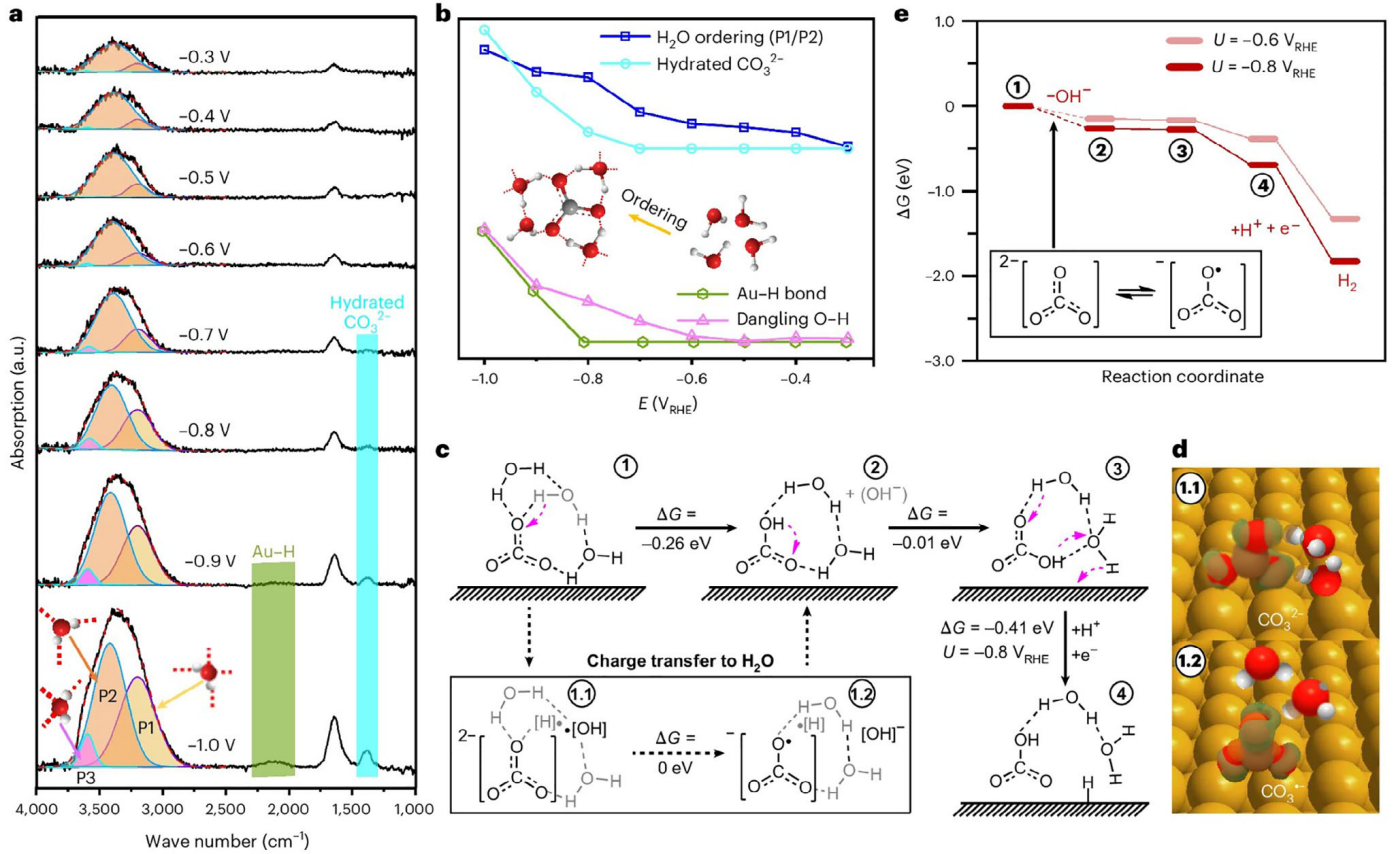
(a) In situ ATR‐SEIRAS of interfacial water at a polycrystalline gold electrode in CO_2_‐saturated 0.1 M KHCO_3_ solution. (b) Corresponding normalized band intensities derived from the spectra in (a). (c) Proposed reaction pathway for Au–H formation via the interfacial water layer on an Au(100) surface. (d) Differential charge density illustrating charge transfer from CO_3_^2−^ and CO_3_•^−^ to interfacial water. Atoms are color‐coded: Au (yellow), C (gray), O (red), H (white). Charge depletion and accumulation regions are shown in blue and orange, respectively. (e) Gibbs energy profile for the HER enhanced by CO_3_•^−^, referenced to the spectrum obtained at 0.3 V vs. RHE. Reproduced with permission from Ref. [129], Copyright 2025, Springer Nature.

For the electrochemical CO_2_RR, the Kley research team discovered that carbonate species in the electrolyte can induce the ordering of interfacial water, thereby improving the reaction pathway and enhancing selectivity. As shown in Figure 22, in situ ATR‐SEIRAS results indicate a peak around 1300 cm^−^^1^, which suggests the presence of hydrated CO_3_^2−^. These radicals form stable hydrated clusters with interfacial water, strengthening the hydrogen‐bonding network among water molecules. Concurrently, the presence of carbonate promotes the ordering of interfacial water, leading to an increase in the proportion of “ice‐like water” (∼3200 cm^−1^). DFT calculations reveal that the hydration energy of the stable hydrogen‐bonded structure formed between CO_3_•^−^ and water molecules is approximately 0.2 eV lower than that of liquid water. Differential charge density analysis further demonstrates that CO_3_•^−^ transfers charge to water molecules, enhancing hydrogen bonding and structural ordering. At high potentials, CO_3_•^−^ acts as a proton relay, facilitating the dissociation of interfacial water into H^+^ and OH^−^. Protons are rapidly transported through the ordered hydrogen‐bonding network to the electrode surface, forming Au‐H bonds and promoting HER. At low potentials, however, carbonate desorption and K^+^ precipitation disrupt the interfacial water structure, hindering proton transfer and allowing CO_2_ reduction to dominate. The authors used the ratio of the peak intensity of ice‐like water near 3200 cm^−1^ (P1) to that of liquid water near 3400 cm^−1^ (P2) as an indicator of water ordering. This ratio showed a positive correlation with the concentration of CO_3_•^−^ [129]. Therefore, the degree of water ordering can be modulated through the design of the electrolyte, including the concentration of carbonate ions and the type of cations, thereby balancing the hydrogen bond network structure, regulating the proportion of adsorbed H on the surface, and enhancing reaction selectivity.

### 5.5 Modulating Lattice Strain

In addition to the strategies discussed above, lattice‐strain engineering has emerged as an additional avenue for modulating interfacial water behavior [130]. Lattice strain can alter atomic spacing and d‐band centers, thereby influencing the adsorption energetics of water molecules and reaction intermediates [131, 132, 133]. For example, Qiao and coworkers proposed a strategy to enhance the performance of seawater electrolysis by tuning interfacial water structure via lattice‐strain engineering. By doping Co into Cu_3_P to form Cu_3‐x_Co_x_P, a compressive strain of 1.4% was introduced. This compressive strain leads to lattice contraction, shortens Cu–Cu bonds, and modifies the electronic structure. ATR‐IR results show that for the doped catalyst, the O–H stretching vibration peak shifts toward higher wavenumbers (blueshift) under negative potentials, indicating enhanced interaction between water molecules and the catalyst surface. In contrast, an undoped sample exhibits a redshift, suggesting stronger hydrogen bonding among water molecules, which is unfavorable for water dissociation. In situ Raman spectroscopy reveals that the proportion of Na^+^‐H_2_O increases with the decreasing potential in the doped catalyst, suggesting that Na^+^‐H_2_O adsorbs more readily onto the surface. In the undoped catalyst, the proportion of 2HB·H_2_O increases while Na^+^‐H_2_O decreases, indicating that water molecules tend to form a strong hydrogen‐bonding network, leading to slower dissociation. Electrochemical tests demonstrate that lattice strain can effectively regulate interfacial water structure and improve HER performance in neutral media [134].

Complementary experimental evidence comes from Zhang et al., who developed biaxially strained MoSe_2_ three‐dimensional nanoshells for alkaline HER [135]. Using operando Raman spectroscopy, they demonstrated that biaxial strain induces a transformation in interfacial water configuration: water adsorption changes from an “O‐down” configuration on Mo to an “O‐horizontal” configuration on ^*^OH via stronger hydrogen bonds. Specifically, the biaxially strained MoSe_2_ showed an increased proportion of K^+^‐H_2_O and an altered hydrogen‐bonding network compared to unstrained samples, which lowered the water dissociation barrier and accelerated HER kinetics.

Notably, Wang et al. provided theoretical evidence for the independent role of lattice strain using DFT and AIMD simulations with explicit water‐solvent environments [136]. By varying only the lattice parameter while keeping other factors fixed, they demonstrated that compressive strain drives interfacial H_2_O into an H‐down configuration on FeP surfaces, whereas tensile strain shifts the orientation to an O‐down configuration with shortened Fe─O bond distances. Importantly, because this study uses computational simulations without experimental confounding factors (e.g., surface defects, compositional changes, or local electric field variations), it provides strong evidence that lattice strain alone can directly influence water molecule orientation and dissociation behavior. Moreover, the tensile strain was found to induce spin polarization in FeP, providing the critical driving force for enhanced alkaline HER kinetics.

It should be noted, however, that lattice strain is often accompanied by correlated effects—such as electronic structure modification (e.g., d‐band center shifts), changes in surface coordination environments and adsorption sites, and variations in local electric fields—making it challenging to unequivocally attribute observed changes in interfacial water configuration solely to strain in experimental systems. Nevertheless, strain represents a tunable parameter that can be intentionally engineered, and growing evidence from both experimental and theoretical studies suggests it plays a non‐negligible role in modulating water adsorption and dissociation behaviors. Future studies combining in situ characterization with strain‐controlled theoretical modeling (e.g., DFT or AIMD with fixed lattice parameters) are needed to further decouple these intertwined factors.

### 5.6 Modulating Catalyst Composition

Jia and coworkers introduced 1,2‐dimethylimidazole (Me‐N_1_C_2_) as an interfacial water structure regulator at the Pt‐H_2_O interface to promote the HER/HOR processes. Their findings revealed that N‐methylimidazole forms strong hydrogen bonds with interfacial water via its pyridine nitrogen, “anchoring” the second layer of water molecules near the Pt surface. This facilitates the diffusion of hydroxide ions at the interface through the Grotthuss mechanism and reduces the energy barrier for water molecule dissociation. In ATR‐SEIRAS, the peak at 3700 cm^−1^ corresponds to the O─H vibration of water molecules. In hydrogen bonds of water molecules, redshift and peak broadening occur for H donors, while no significant changes are observed for H acceptors. Thus, the peak at 3000 cm^−1^ corresponds to the strong hydrogen‐bonded water structure containing H donors near the Pt surface, whereas the sharp peak at 3500 cm^−1^ originates from the weak hydrogen‐bonded water structure composed of H acceptors. Typically, as the potential decreases, the intensity of the peak near 3000 cm^−1^ gradually weakens, while there is a slight increase between 3300 and 3500 cm^−1^, and both bands undergo a blueshift. These spectral changes are attributed to the potential‐dependent reorientation of H‐up water (proton donors) to H‐down water (proton acceptors). However, the results show that after adding Me‐N_1_C_2_, the strong hydrogen‐bonded structure of interfacial water no longer undergoes blueshift or weakening as the potential decreases, indicating that Me‐N_1_C_2_ stabilizes the hydrogen‐bond network of interfacial water. Calculation results demonstrate that Me‐N_1_C_2_ molecules adsorb parallel to the Pt surface (confirmed by Raman spectroscopy) and pull water molecules closer to the interface through N_3_─H_2_O bonds. By stabilizing the second layer of water molecules, N‐methylimidazole reconstructs the hydrogen‐bond network disrupted by the interfacial electric field, providing a rapid diffusion channel for hydroxide ions [54].

The intrinsic dependence of electrocatalytic activity on electrode material originates, to a large extent, from the material‑dependent PZC. The PZC determines the charge state of the electrode surface at a given applied potential, thereby governing the structure of the EDL—including the orientation, hydrogen‑bonding network, and rigidity of interfacial water molecules. Consequently, modulating catalyst composition (e.g., through alloying, doping, or surface modification) provides a powerful strategy to tune the PZC, reshape the EDL, and ultimately optimize interfacial water configurations for enhanced reaction kinetics [15]. For example, Koper group proposed that Ni(OH)_2_ shifts the PZC toward the hydrogen region, thereby reducing the reorganization energy of interfacial water and facilitating the transport of OH^−^ across the EDL [28]. Sarabia et al. established a correlation between Ni coverage and PZC through CO displacement experiments. Using laser‐induced temperature jump experiments, they observed that as the coverage of Ni(OH) _2_ increases, the degree of ordering of interfacial water molecules significantly decreases in the low‐potential region where HER occurs (0.1–0.4 V vs. RHE). Specifically, the water structure shifts from a highly oriented “H‐down” configuration to a more disordered state. This phenomenon was attributed to the inverse shift of PZC/PME induced by Ni(OH)_2_, which weakens the interfacial electric field strength, diminishes the field‐driven alignment of water molecules, reduces the energy required for water reorganization, and thereby promotes water dissociation. The study further found that as Ni(OH)_2_ coverage increases, the transient laser signal shifts from negative to positive, indicating an inversion in water configurations [47].

Wei and coworkers modified Pt/C catalysts with metal oxides to regulate the configurations of surface water. They found that on the Pt surface, water molecules tend to adsorb in an H‐up configuration, which is unfavorable for water dissociation. In contrast, after modification with TiO_2_, water molecules form stronger interactions with it and tend to arrange in an H‐down configuration. Compared with Pt (111) −KOH, the close distance of H_2_O−TiO_2_ and the orientation of interfacial water are more conducive to the continuous activation of water to form adsorbed ^*^H, which facilitates water dissociation. The authors attribute this phenomenon to the high dielectric constants of metal oxides, which generate stronger local electric fields under cathodic polarization. These enhanced fields promote the enrichment of highly hydrated potassium ions and induce water molecules to adopt the H‐down orientation, thereby reducing the energy barrier for water dissociation. This finding underscores that adjusting the catalyst composition can effectively modulate interfacial water configurations [137].

## 6 Conclusions and Outlooks

**FIGURE 23:**
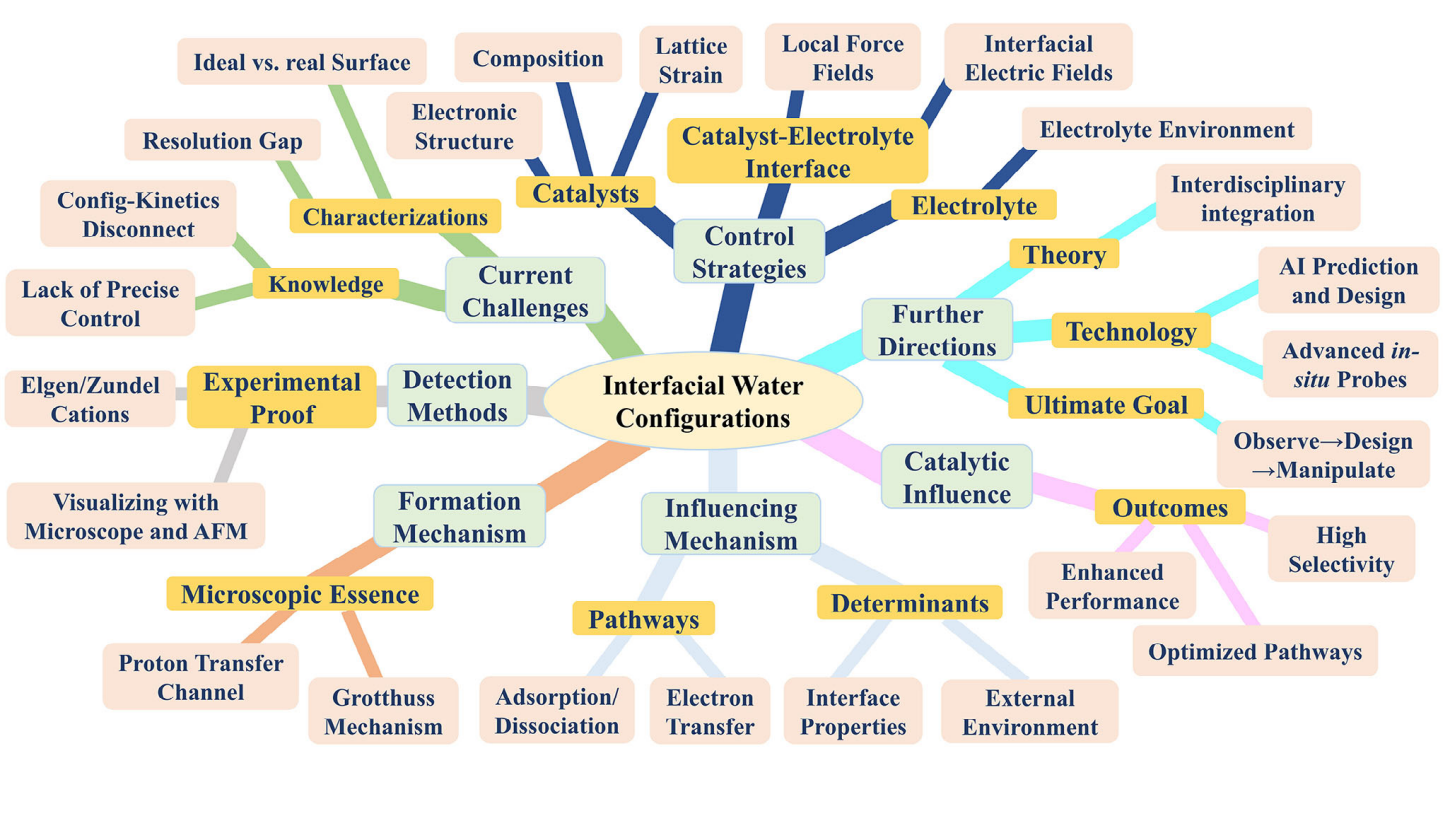
The mind map summarizes the key points associated with interfacial water configurations.

In summary, interfacial water is far from being a passive “spectator.” Its diverse configurations are both a direct reflection and a critical regulator of interfacial physicochemical properties. This review systematically outlines the various forms of interfacial water configurations, their origins, detection methods, profound effects on catalytic reactions, and strategies for their active modulation. The key points associated with interfacial water configurations are summarized in the mind map shown in Figure 23. Complex factors influence the evolution of water configurations, but the understanding of these effects has broad applications. Understanding interfacial water is crucial for studying electrocatalytic mechanisms. We identified patterns in the evolution of water configurations across various electrochemical reactions, extending from passive observation to active manipulation of interfacial water, thereby realizing the vision of designing highly active and selective electrochemical processes. Studies indicated that water configurations are dynamically determined by the intrinsic properties of the interface as well as external environmental conditions. Generally, interfacial water with fewer intermolecular hydrogen bond configurations is more prone to dissociation. Their role in promoting catalytic reactions can be elucidated by integrating in situ spectroscopy with theoretical simulations. The hydrophilicity/hydrophobicity and defect structures of electrode surfaces can alter the configuration of adsorbed water molecules. Meanwhile, lattice strain induced by methods such as doping modifies the interfacial force field, thereby similarly influencing interfacial water arrangement. Under the influence of an interfacial electric field, the oriented alignment of water molecules affects electron transfer, which in turn governs the adsorption and dissociation behavior of water at catalytically active sites. The synergistic interplay of these factors clearly demonstrates that specific water configurations can optimize reaction pathways, accelerate mass transfer, increase selectivity, and lower reaction energy barriers, thereby significantly enhancing catalytic performance.

Based on the discussions in this review, the following conclusions can be clearly drawn: In numerous water‐involved reactions, although the hydrogen‐bonding environments differ, the system's characteristics are governed by proton diffusion, which is influenced by changes in interfacial properties, external environmental conditions, and strain variations. In simpler terms, the transitions among 4HB‐H_2_O, 2HB‐H_2_O, and free water serve as channels for the transfer and adsorption of the reaction intermediate H, becoming crucial to the reaction. Designing interfacial structures that meet specific requirements through modification and other means can optimize surface reorganization, reduce the adsorption energy of intermediates, and enhance reaction selectivity, thereby forming the core of improving reaction efficiency. Regarding the fundamental influence of water configurations on catalytic reactions, earlier studies have shown that hydrogen‐bond hopping exchange controls water dynamics and is critical to proton transport in acidic and basic aqueous solutions. Among the various contributing pathways, the Grotthuss mechanism represents a particularly important one for interfacial water effects, though it is not the sole contributor; other mechanisms such as direct water dissociation and electrode‐involved proton transfer pathways also play significant roles depending on the specific interfacial conditions [138]. In this context, proton diffusion in water is a multi‐step process limited by two consecutive stable hydrogen‐bond exchanges, and a key aspect is the reformation of a hydrogen bond at the previous proton donor site after proton transfer [139]. Related water configuration changes are mostly characterized by vibrational spectroscopy, while theoretical analysis of proton transport mechanisms still primarily relies on simulation calculations. Rare direct experimental insights include the work by Tian et al., who used cryogenic atomic force microscopy under ultra‐high vacuum to observe hydrated proton motifs (Eigen cations, H_3_O^+^(H_2_O)_3_, and Zundel cations, H_5_O_2_^+^) on metal surfaces. It should be clarified that these hydrated‐proton structures are distinct from the hydrogen‐bond coordination states of neutral water molecules (e.g., 4HB‐H_2_O and 2HB‐H_2_O), though both concepts are important for understanding interfacial water structure [48]. Based on the analysis of proton transport pathways, numerous studies have achieved the goal of designing interfacial water structures through interface modification and functionalization, significantly enhancing both the activity and selectivity of catalytic reactions.

Although multi‐scale, multi‐technique approaches have been employed to reveal the configurational changes of interfacial water, numerous practical challenges remain unresolved in this field. For instance, the atomic force microscopy technique mentioned above, which directly observes Eigen‐ and Zundel‐type hydrated protons on surfaces such as Au (111) and Pt (111), exhibits significantly weaker signals on Au than on Pt. This indicates that even meticulously designed single‐crystal surfaces can still yield unsatisfactory signals, raising concerns about the feasibility of probing real catalytic surfaces with complex, multi‐factor coupling effects. However, if such techniques cannot be applied to practical catalytic interfaces, the true dynamic processes of reactions cannot be directly observed.

Additionally, most characterization methods provide temporally or spatially averaged information, making it difficult to capture the dynamic fluctuations of water molecules across timescales ranging from femtoseconds to seconds and spatial scales from nanometers to micrometers. Regarding volcano‐type relationships, the often‐cited principle that moderate H adsorption favors reaction selectivity typically relies on empirical judgment or exhaustive trial‐and‐error [140]. Current research still lacks the ability to establish quantitative correlations, and there remains an absence of precise correspondence between water configurations and reaction kinetics.

Therefore, the current state of the field still falls short of achieving precise control over interfacial water structures. This limitation primarily manifests in the multiple challenges spanning from dynamic monitoring to accurate manipulation. In the future, deciphering the transient fluctuations of water configurations in real reactions will depend on advances in in situ technologies with higher spatiotemporal resolution, combined with in‐depth, AI‐enabled cross‐scale computational methods.

From a technology‐driven perspective: Future efforts should focus on developing more powerful in situ techniques. This includes spectroscopy and imaging technologies with full spatiotemporal resolution, high signal‐to‐noise ratios, and structural sensitivity, enabling observations under true working conditions. From a theoretical‐deepening perspective: Greater emphasis should be placed on interdisciplinary collaboration, incorporating fields such as condensed matter physics. This will aid in studying electronic states, phonon modes, and other properties to better understand the role of water configurations in energy transfer and transformation. From a future‐development perspective: The full potential of artificial intelligence should be leveraged. By integrating high‐throughput computing and big data analytics, more accurate reaction environment models can be constructed to predict optimal water configurations.

Thus, a profound understanding and precise control of interfacial water configurations represent not only a core scientific frontier for unveiling the origins of heterogeneous catalysis but also a critical breakthrough for designing next‐generation, high‐performance catalytic systems and enabling green energy and chemical transformations. It is anticipated that new tools and paradigms will be developed to ultimately achieve the leap from “observing water” to “designing water” and, finally, to “manipulating water”. We believe that interdisciplinary collaboration can shift research from passive observation to active design, ultimately enabling the customization of catalytic interfaces through water‐structure interface engineering. This will provide novel solutions for efficient energy conversion and green synthesis.

## Author Contributions

**Lin Guo**: funding acquisition, conceptualization, methodology, investigation, supervision. **Chaoyu Li**: writing – review and editing, investigation. **Yanxia Yuan**: data curation, writing – review and editing. **Aoqi Wang**: conceptualization, methodology, data curation, investigation, formal analysis, validation, writing – original draft. **Yang Song**: conceptualization, methodology, formal analysis, writing – review and editing, supervision. **Chenchen Weng**: conceptualization, methodology, validation, formal analysis, writing – review and editing. **Wei Lin**: conceptualization, methodology, investigation, supervision, funding acquisition, project administration, visualization, validation, writing – review and editing. **Xue Yang**: conceptualization, methodology, investigation, formal analysis, supervision, writing – review and editing, project administration, funding acquisition.

## Conflicts of Interest

The authors declare no conflicts of interest.

## Data Availability

Data sharing not applicable to this article as no datasets were generated or analysed during the current study.
